# Metabolic modelling-based in silico drug target prediction identifies six novel repurposable drugs for melanoma

**DOI:** 10.1038/s41419-023-05955-1

**Published:** 2023-07-26

**Authors:** Tamara Bintener, Maria Pires Pacheco, Demetra Philippidou, Christiane Margue, Ali Kishk, Greta Del Mistro, Luca Di Leo, Maria Moscardó Garcia, Rashi Halder, Lasse Sinkkonen, Daniela De Zio, Stephanie Kreis, Dagmar Kulms, Thomas Sauter

**Affiliations:** 1 Department of Life Sciences and Medicine, University of Luxembourg, Belvaux, Luxembourg; 2 Experimental Dermatology, Department of Dermatology, TU-Dresden, Dresden, Germany; 3 National Center for Tumour Diseases, TU-Dresden, Dresden, Germany; 4 Melanoma Research Team, Danish Cancer Society Research Center, Copenhagen, Denmark; 5 Luxembourg Centre for Systems Biomedicine, University of Luxembourg, Belvaux, Luxembourg; 6 Department of Drug Design and Pharmacology, Faculty of Health and Medical Sciences, University of Copenhagen, Copenhagen, Denmark

**Keywords:** Cancer metabolism, Virtual screening

## Abstract

Despite high initial response rates to targeted kinase inhibitors, the majority of patients suffering from metastatic melanoma present with high relapse rates, demanding for alternative therapeutic options. We have previously developed a drug repurposing workflow to identify metabolic drug targets that, if depleted, inhibit the growth of cancer cells without harming healthy tissues. In the current study, we have applied a refined version of the workflow to specifically predict both, common essential genes across various cancer types, and melanoma-specific essential genes that could potentially be used as drug targets for melanoma treatment. The in silico single gene deletion step was adapted to simulate the knock-out of all targets of a drug on an objective function such as growth or energy balance. Based on publicly available, and in-house, large-scale transcriptomic data metabolic models for melanoma were reconstructed enabling the prediction of 28 candidate drugs and estimating their respective efficacy. Twelve highly efficacious drugs with low half-maximal inhibitory concentration values for the treatment of other cancers, which are not yet approved for melanoma treatment, were used for in vitro validation using melanoma cell lines. Combination of the top 4 out of 6 promising candidate drugs with BRAF or MEK inhibitors, partially showed synergistic growth inhibition compared to individual BRAF/MEK inhibition. Hence, the repurposing of drugs may enable an increase in therapeutic options e.g., for non-responders or upon acquired resistance to conventional melanoma treatments.

## Introduction

Deregulation of two major signalling pathways, the RAS-RAF-MEK-ERK and PI3K-AKT-PTEN, are key drivers of melanoma development and progression [1], with ~50% and ~25% of patients expressing constitutively active mutants of MAP-kinases BRAF and NRAS, respectively [2]. Combined targeted inhibition of mutated BRAF and downstream MEK kinases, or alternatively immune checkpoint inhibition, currently provide good therapeutic options for the systemic treatment of melanoma, offering a long-term survival to ~30% of the patients. Unfortunately, relapse rates to targeted kinase inhibition are high, still causing the death of ~70% of melanoma patients suffering from an advanced melanoma stage due to enhanced re-growth of treatment-resistant metastases [3]. To date, intervention strategies are based on the individual mutation status of the BRAF and NRAS oncogenes. However, other pathophysiological modifications within melanoma cells may contribute to therapy resistance [4, 5], demanding for large-scale computational models to simulate the complex physiology of a tumour in an integrated manner and comprehensively foster the identification of alternative therapeutic vulnerabilities applying high throughput in silico approaches [6, 7].

In melanoma, the oncogenic mutation of BRAF promotes metabolic reprogramming [8, 9], thereby often favouring glycolysis for energy production. Moreover, BRAF inhibition (BRAFi)-resistant melanoma cells were shown to present with deregulation of the fatty acid synthase (FASN) [10], which was proposed as a metabolic target for melanoma, prostate, and breast cancer treatment [11]. Accordingly, statins have been investigated as alternative treatment options for melanoma [12, 13], and other cancer types [14, 15]. These drugs act by limiting the availability of lipids and consequently reducing proliferation [12, 13]. In this context, lovastatin, a β-Hydroxy β-methylglutaryl-coenzyme A reductase (HMGCR) inhibitor, was shown to induce apoptosis in several BRAF-mutated and metastases-derived melanoma cell lines [16]. Furthermore, slow-cycling tumour cells are addicted to glutamine-fuelled oxidative phosphorylation (OXPHOS) and rely on lipidome adaptations, particularly fatty acid oxidation pathways [17] for cell migration and invasion [18].

To describe and analyse metabolism in general and metabolic alterations specifically at a genome-scale level, metabolic network modelling using constraint-based approaches has been successfully applied for various cancer [19, 20] and many other diseases, e.g. COVID-19 [21]. The respective mathematical models can be utilised for identifying sensitive network targets as well as drug candidates for repurposing [19]. Thereby, metabolic modelling allows predicting cancer-specific targets required to sustain higher proliferation rates as well as pronounced systemic migration. Drugs shutting down these metabolic targets alone or in combination with currently applied therapeutics, have the potential to deprive fast-cycling cells of nucleotides and lipids required to gain increase in tumour mass [19]. Hence, drugs affecting nitric oxide homeostasis and production (NO-based drugs), that showed promising results for metastatic melanoma patients, might extend the panel of treatment options for non-responders to current therapies and relapse cases [22].

To capture cancer-specific metabolic alterations on a genome scale, and to examine how these alterations could be exploited to derive novel melanoma treatment regimens, we have reconstructed over 10,000 metabolic models, and computationally identified 54 putative gene targets and 12 drug candidates for melanoma treatment. We compared our candidate drugs to known anti-melanoma and NO-based drugs using publicly available drugs screens and high-throughput CRISPR data and experimentally validated six of these candidates in BRAF and NRAS mutated cell lines in mono and combination treatment with BRAF/MEK inhibitors.

## Material and methods

### Cell culture (IN-HOUSE dataset and validation)

See Supplementary Methods (Supplementary File 1).

### Data

For the present study, publicly available RNA-seq data from The Cancer Genome Atlas programme (TCGA patient data (GEO: GSE62944) [23], and the Cancer Cell Line Encyclopaedia (CCLE)) were combined with IN-HOUSE generated RNAseq data according to Table 1 and Supplementary File 1.

**Table 1:** Overview of the data sets, their characteristics, and the respective number of models which were built.

Dataset	Type	Number of samples	Number of replicates	Description	Number of models (consensus/sample-specific)
Data					Models
Cancer					
TCGA	23 Cancer types patients	8792	mostly 1	RNA-seq data of tumour samples from various cancer types of TCGA consortium reprocessed by ref. [33]	23/8792
SKCM	Melanoma patients	472	1	RNA-seq data of SKCM tumour samples of TCGA consortium reprocessed by [33] from different stages	1/472
IN-HOUSE	Melanoma cell lines	9 cell lines (28 samples)	3–4	RNA-seq data of the A375, A375IZI, MALME 3 M, WM1346, WM1366, MeWo and SK-Mel5 melnaoma cell lines, and TUMEL patient-derived melanoma isolates	9/28
CCLE	Melanoma cell lines	49	1	RNA-seq data from 49 cell lines of CCLE collection	−/49
Control					
TCGA-CONTROL	Healthy control tissues for 19 cancer types patients	740	1	RNA-seq data from the TCGA consortium of samples taken from 19 normal tissues near the tumour	19/740
TCGA-CONTROL-LIVER	Liver control patients	50	1	RNA-seq data from the TCGA consortium of liver samples near a LIHC tumour	1/50
TCGA-CONTROL-KIDNEY	Kidney control patients	129	1	RNA-seq data from the TCGA consortium of kidney samples KIRC, KIRP, KICH tumours	3/129
IN-HOUSE-CONTROL	Melanocytes	1 cell line (3 samples)	3	RNA-seq data from melanocytes	1/3

See “Material and methods” section for more details on the used cell lines and growth conditions.

### Models

**Fig. 1:**
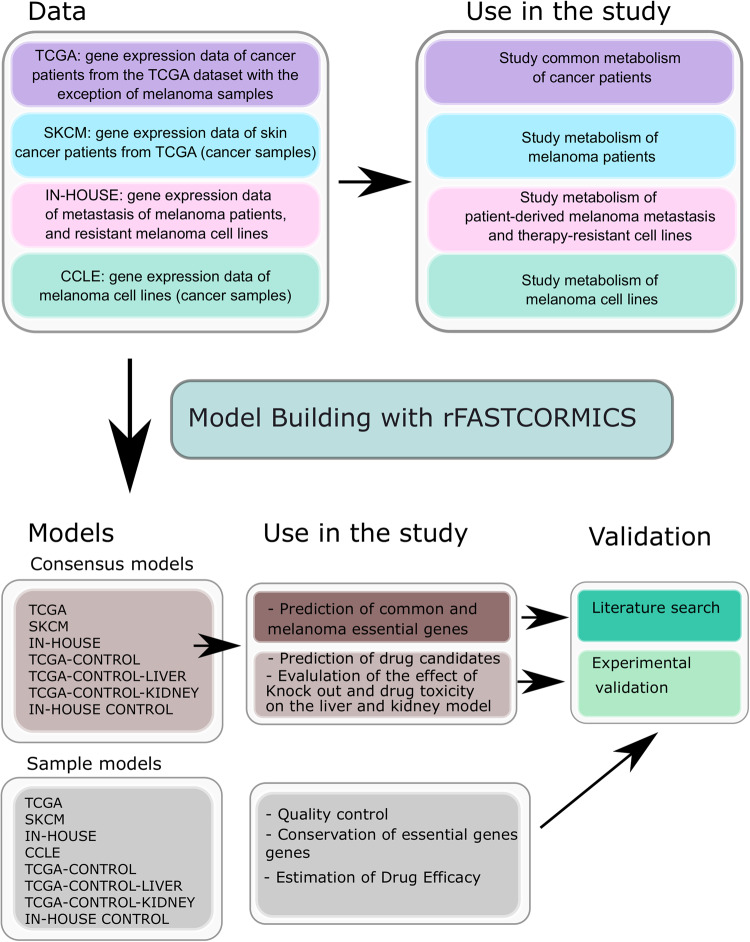
In silico knock-outs across various melanoma datasets allow pinpointing common metabolic targets. Melanoma cell line gene expression data from CCLE, and melanoma patient gene expression data from the TCGA (SKCM) and an in-house dataset (IN-HOUSE) composed of melanocytes, melanoma metastatic patient samples, resistant and sensitive melanoma cell lines were used to reconstruct melanoma cell line and patient sample and consensus models. Single in silico knock-outs and drug deletion were performed to identify cancer-common essential genes, and candidate drugs that are common between patient- and cell line-derived data. The predicted essential genes were validated against existing knowledge and publicly available CRISPR high-throughput screens and the most promising drug candidates were validated in vitro.

The metabolic models were reconstructed with a member of the FASTCORE family [24, 25]: the rFASTCORMICS workflow [19] (https://github.com/sysbiolux/rFASTCORMICS) (Fig. 1 and Table 1**)** using Recon 2.04 as input reconstruction, RPMI composition as medium constraint and the biomass function and ATP maintenance as objective functions. Recon 2.04 was chosen as it outperformed Recon3D [26] for essential genes prediction in previous studies [27].

Two types of models were built: (1) sample-specific models, where each RNA-seq sample was used individually to reconstruct a model representing the metabolism of the given sample. (2) (a) A consensus model of samples originating from the same condition. Samples from one condition were pooled together and only reactions being active in 90% of the samples were considered for reconstruction. (b) A consensus model of cell lines, in which replicates were pooled to obtain a cell line model. See Table 1 for the numbers on sample-specific and consensus models. As the raw data from the TCGA and CCLE were not easily accessible, the analyses were performed on the processed data for these datasets. Consequently, due to batch effects, the three datasets were considered independently.

Consensus models per cancer type were built to predict essential genes that could potentially serve as drug targets. Sample-specific models were used as quality control (Supplementary Figs. 1 and 2), to assess the drug efficacy and predict the population of patients responding to a given drug. More specifically, RNA-seq data from skin cancer patient samples (Skin Cutaneous Melanoma, SKCM) of TCGA and melanoma cell lines from CCLE were employed to reconstruct metabolic models and identify melanoma-specific essential genes arising from metabolic rewiring. Furthermore, data of melanoma metastasis (IN-HOUSE), melanocytes (IN-HOUSE-CONTROL), and different drug-sensitive and resistant melanoma cell lines, were used to assess the effect of the predicted essential genes in advanced stages of melanoma.

Patients’ data derived from other cancer types within TCGA database were included to distinguish between genes commonly dysregulated in cancer, and melanoma-specific essential genes. Finally, models of healthy control including liver (TCGA-CONTROL-LIVER) and kidney (TCGA-CONTROL-KIDNEY) samples were built to assess the potential negative impact of the respective drug on essential healthy tissues (Fig. 1).

### Gene essential analysis and in silico drug prediction

In silico essential drug prediction was performed on all models using a modified version of the singleGeneDeletion function from the COBRA toolbox [28] and the biomass and ATP production as an objective function for cancer and control models, respectively. For the consensus model, a gene was considered essential if the growth ratio between the wild type and the knock-out was below 50% for the cancer and at least 90% for the healthy models.

For the sample-specific models, the same growth ratios needed to be observed in at least 50% of the cancer and 10% of the healthy models to consider a gene to be essential.

In parallel, the effect of the drugs were simulated using an adapted version of the singleGeneDeletion of the COBRA toolbox [28] (Drug Deletion function): Therefore, DrugBank v5.1.3 [29] was mined to identify 1175 drugs inhibiting metabolic genes (genes present in Recon 2.04) and their targets that were then used as input for the Drug Deletion function. The targets were mapped to the model genes and based on the gene-protein-reaction (GPR) rules, the associated reactions were inactivated by setting their bounds to 0. The thresholds from the essential gene analysis were used to find drugs with an effect on the cancer biomass but not on healthy ATP models.

For the sample-specific models per dataset, a drug essentiality score was calculated for each drug by determining the number of samples in which the drug reduced the biomass or the ATP production below the desired threshold for cancer and healthy models, respectively. A drug essentiality score of 1 signifies that the drug shuts down essential genes in 100% of the samples. Enrichment scores for cancer drugs were calculated (as described in ref. [19]).

### Drug prioritisation

See Supplementary Methods (Supplementary File 1).

### Experimental validation

Dose response curves and determination of IC_50_ values were assessed for the 12 candidate drugs using 3-fold dilution series (see Supplementary Methods (Supplementary File 1) for more details). Additionally, four drugs (cladribine, gemcitabine, lovastatin and fluvastatin) were combined with 1 µM palbociclib for their IC_50_ determination. Cell viability was assessed with the PrestoBlue Cell Viability Reagent (ThermoFisher Scientific) on a Cytation 5 (Biotek). Determination of IC_50_ was performed as described before [30].

Synergy tests with SynergyFinder: melanoma cells were treated with a combination of BRAF, MEK- and CDK- inhibitors at 8 concentrations (in a 1:2 or 1:1.5 dilution range) based on their respective IC_50_ values. Synergy scoring was performed as published before [30]. Zero Interaction Potency (ZIP) scores <−10 and >10 correspond to an antagonist and synergetic effect, respectively. For details on cell viability, proliferation, Propidium Iodide (PI) dead cell staining and caspase 3/7 Ac-DEVD-AFC apoptosis assays, see Supplementary Methods (Supplementary File 1**)**.

## Results

### Cancer cells, including melanoma, depend on de novo metabolic synthesis pathways to sustain high proliferation levels

**Fig. 2:**
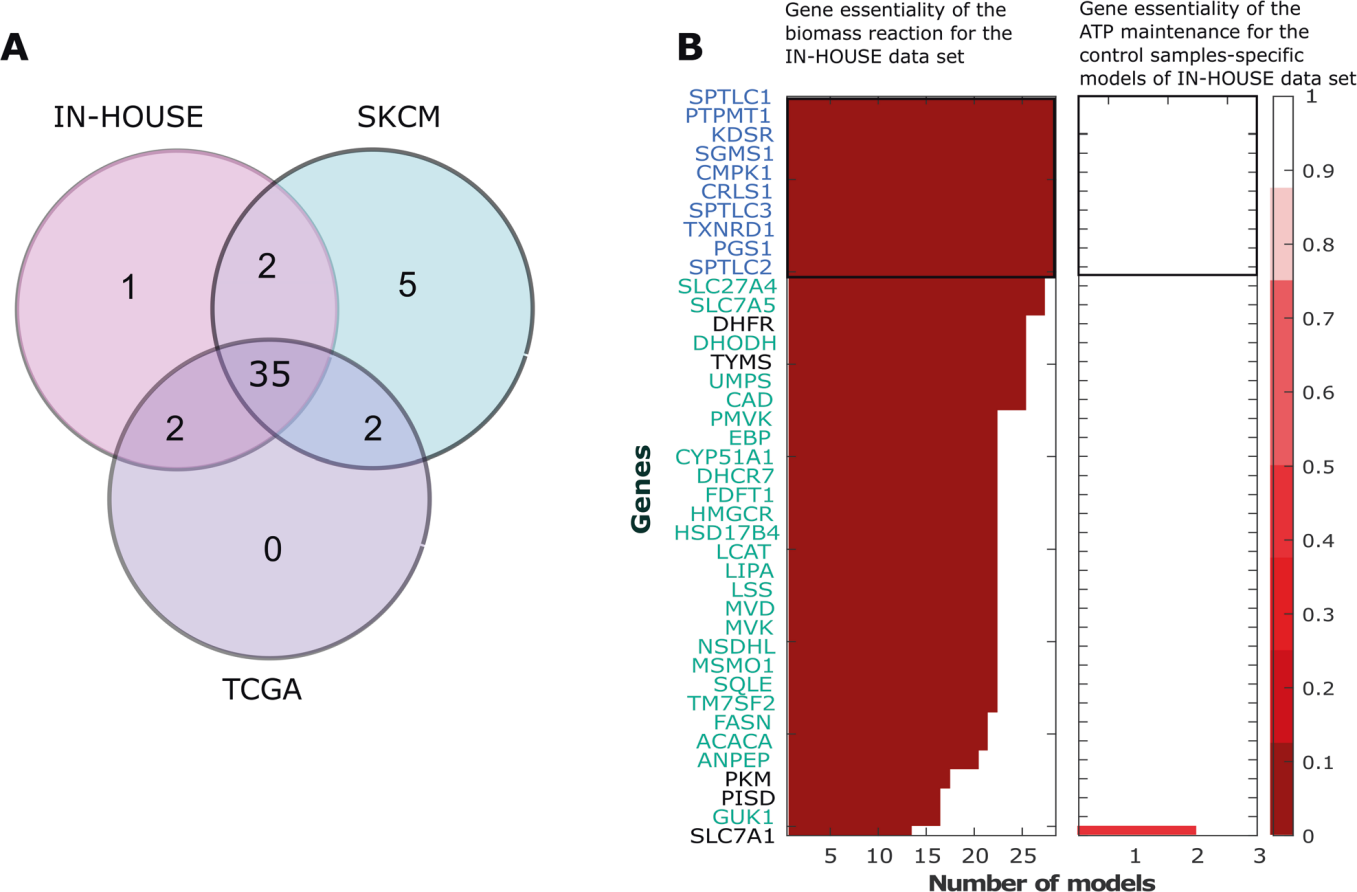
Essential genes across multiple melanoma and cancer cell types are promising drug targets. In silico knock-outs were performed to identify vulnerabilities present in most melanoma samples. **A** Venn diagram presenting essential genes among TCGA, SKCM and IN-HOUSE models. **B** In silico gene deletion analysis on the sample-specific IN-HOUSE models that shows the number of models that are affected by the deleted gene. The colour code indicates the ratio of the predicted biomass in knock-out vs. wild-type, with dark red indicating a fully effective and white a non-effective knock-out. The *y*-axis represents the IN-HOUSE essential genes sorted by efficacy (fraction of affected models). The upper part of the left panel includes genes (in blue) whose deletion disables the biomass reaction in all of the 28 cancer models of the IN-HOUSE dataset. Below are genes (in green) that disable the biomass reaction in most cancer models but not all. The effect of gene deletion on the ATP production of the monocytes (IN-HOUSE-CONTROL) models is depicted in the right panel. Genes in black are not part of the shared 35 genes predicted by the consensus models.

Consensus metabolic networks were reconstructed at the genome-scale for the TCGA, SKCM and IN-HOUSE datasets to capture metabolic alterations in cancer in general and in melanoma specifically. The median number of reactions in the respective models were 1780, 1773 and 1686, respectively. Applying in silico essentiality analysis, 39 genes were predicted to reduce the biomass production by at least 50% in the TCGA, 44 in SKCM and 40 in IN-HOUSE models, with 35 essential genes being shared between all three datasets (Fig. 2A). This suggests the existence of common essential genes across all cancer types being indicative of commonly implemented metabolic rewiring strategies. In all, 13 out of the 35 predicted common essential genes were shown to be involved in the cholesterol biosynthesis pathway, 1 in cardiolipin synthesis, 2 in glycerophospholipids, 5 in sphingolipid metabolism, 6 in de novo synthesis of nucleotides (CMPK1, TXNRD1, CAD, DHODH, UMPS, GUK1), and 5 genes in de novo synthesis of fatty acids (ACACA, LCAT, LIPA, FASN HSD17B4), respectively (Supplementary Table 2). Noteworthy, fotemustine that is approved in some countries against melanoma brain metastasis, inhibits Thioredoxin Reductase 1 (TXNRD1) [31]. The three remaining genes represented two solute carrier transporters (SLC27A1 and SLC7A5) for amino acids [32] and fatty acids and one membrane bound protease (ANPEP) that plays a role in tumour invasion and metastasis [33].

Only one gene, namely RPIA (coding for the Ribose-5-phosphate isomerase), required for ATP maintenance according to the TCGA and SCKM models, was also found to be essential in the TCGA-CONTROL-LIVER model (Supplementary Fig. 4) and hence no side-effects on the liver metabolism were predicted by our analysis.

We further assessed if the genes predicted with the consensus models are likely to be effective in inducing cell death in different melanoma backgrounds. However, as no clear separation could be observed in a principal component analysis between the metabolic gene expression of the primary and metastatic samples in the TCGA and CCLE samples (Supplementary Fig. 3), the respective models could not be used to identify metastatic specific metabolic alterations.

Thus, knock-out of metabolic genes (mainly the 35 genes predicted to be essential by the consensus models) was performed utilising the IN-HOUSE (Fig. 2B) and TCGA, SKCM and CCLE sample-specific models (Supplementary Figs. 5 and 6) that have a higher sensitivity as being reconstructed from one sample than consensus models that are obtained after pooling all the samples of a condition. This allows predicting the drug efficacy in these various patients and cell line backgrounds and estimating the number of patients needed to be treated before seeing a positive outcome (Number Needed to Treat, NNT). As control, knock-outs of our predicted targets were performed in healthy melanocyte sample-specific models (IN-HOUSE-CONTROL) to assess the impact on ATP maintenance (Fig. 2B).

Out of the 35 predicted essential genes (Fig. 2A), the knock-out of 10 of these genes completely shut down biomass production in all 28 melanoma sample-specific models within the IN-HOUSE dataset (Fig. 2B left, in blue), as well as TCGA, SKCM and CCLE models (Supplementary Figs. 5 and 6), without affecting the ATP production in the IN-HOUSE-CONTROL sample-specific models (Fig. 2B right), and only minimally affected the ATP production of the TCGA-CONTROL dataset. The gene products of these top ten genes include enzymes implicated in the de novo synthesis of nucleotides, cardiolipins, and glycerophospholipid metabolism and sphingolipid metabolism (Supplementary Table 2).

Another set of essential genes (Fig. 2B left, in green, Supplementary Table 2) reduced the biomass production to zero in most - but not all - IN-HOUSE models without significantly affecting the control models (Fig. 2B right) and are partially shared among all cancer models (Supplementary Figs. 5 and 6). These genes mainly regulate the cholesterol, and fatty acid metabolism and biosynthesis, and the nucleotide interconversion which is coherent with the need to generate lipids and nucleotides in fast-cycling cells. Thus, these genes are less likely to affect cancer cells with a lower proliferation, explaining the lack of response in some models (Fig. 2B left, in green). Other predicted essential genes (PKM2, PISD) are linked to oxidative phosphorylation (OXPHOS). Additionally, in an extended list (top 100) based on the INHOUSE, CCLE and SKCM sample-specific models (Supplementary Figs. 6 and 7, in purple), another large set of 20 essential genes was identified, which are involved in controlling the final steps within the respiratory electron transport chain and the ubiquinol-6 cytochrome C reductase (CYOR u10m) to different degrees. This strong dependency on OXPHOS suggests an enrichment of slow-cycling cells [17, 18] in the IN-HOUSE and to some extent in SKCM and CCLE datasets. These data turned the switch between glycolysis and OXPHOS to a relevant topic for further investigation.

Taken together, the predicted 35 common essential genes (Fig. 2A) are part of metabolic pathways (mostly of lipids, carbohydrates and amino acids) required to sustain high proliferation rates. These genes were predicted to be essential across all tested datasets, making them promising drug targets across different melanoma backgrounds.

### 12 candidate drugs, among them cladribine, gemcitabine, lovastatin and tamoxifen, are predicted to be efficient for most cancer types as targeting common vulnerabilities of cancer cells

Like gene essentiality analysis, drug target deletion in cancer and healthy control models was used to identify drugs reducing biomass production, and thus proliferation specifically of cancer cells without affecting healthy cells. Furthermore, it allows identifying drugs that are synthetically lethal by targeting different metabolic branches.

**Fig. 3:**
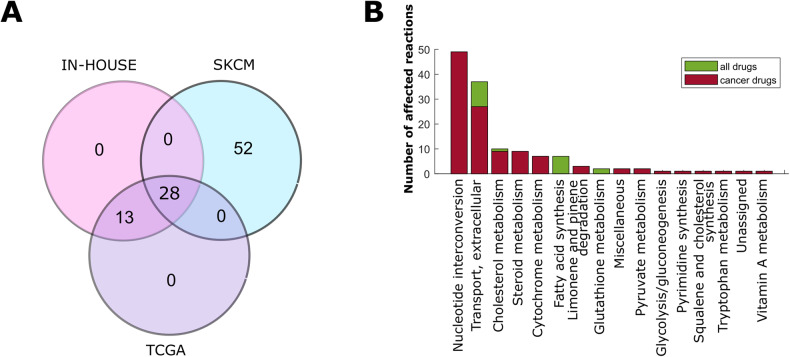
28 drugs targeting biosynthetic pathways are predicted to reduce growth in all melanoma datasets. **A** 28 predicted drugs are shared between IN-HOUSE, TCGA, SKCM consensus models . **B** Out of the 28 drugs, 12 have been marked as anticancer drugs by at least one database (Supplementary Table 3). These 12 drugs have many diverse targets in a variety of different pathways as defined in Recon 2.04.

Applying this drug deletion pipeline on the consensus IN-HOUSE models, 41 out of around 3000 DrugBank-retrieved FDA-approved drugs were predicted to reduce melanoma growth rate below 50% (Fig. 3A), without affecting primary melanocytes (IN-HOUSE-CONTROL), TCGA-CONTROL-LIVER or TCGA-CONTROL-KIDNEY models. However, these 41 drugs were also found effective in affecting other than melanoma cancer models within the TCGA database (Fig. 3A). These drugs predicted by the consensus models were also found essential throughout at least 50% of the sample-specific models, suggesting that these are likely to have an impact on broad melanoma backgrounds (Supplementary Figs. 8–10).

Nevertheless, 28 out of these 41 drugs were also predicted to be effective in the SKCM model, thus representing promising treatment options particularly for melanoma (Fig. 3A). Significant enrichment of this set of 28 drugs, already being in use for cancer treatment, was found across various databases like SEER (https://seer.cancer.gov/), Cancer GOV (https://www.cancer.gov/) and centerwatch (https://www.centerwatch.com/; Supplementary Table 3). Furthermore, the 28 drugs revealed 54 known metabolic targets in the generic metabolic reconstruction Recon 2.04 and proved to be responsible for the inhibition of 134 reactions across 16 pathways (Fig. 3B). 49 of these reactions occur in the nucleotide interconversion pathway as well as additional targets within the extracellular transport, and the cholesterol and fatty acid synthesis pathways. The latter is not yet targeted by approved drugs according to currently available databases and might therefore be interesting for drug repurposing efforts.

**Fig. 4:**
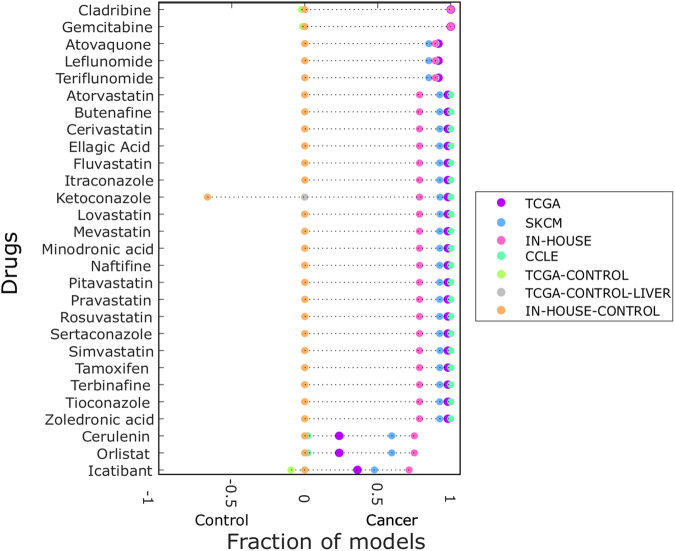
25 out of 28 drugs are predicted to reduce growth of over 70% of the sample models. The fraction of sample-specific models per dataset predicted to respond to the 28 candidate drugs has been determined for the cancer models (TCGA, SKCM, IN-HOUSE, CCLE, right-hand-side) and control models (left-hand-side). Drugs were sorted by their efficacy on the IN-HOUSE dataset. On the *x*-axis the fractions of the models are shown that responded to the drugs in the y-axis. These fractions are estimates for the respective drug efficacy ranging from 0 (not efficacious) to 1 (fully efficacious). For the control, a fraction to −1 indicates a prediction of adverse effects on ATP maintenance in the control samples, while fractions close to 0 indicates no such effects.

To further consolidate the most promising drugs regarding melanoma treatment, their efficacy was predicted on the sample-specific TCGA, SKCM, IN-HOUSE and CCLE models, as well as on the control sample-specific models (Fig. 4). Overall high efficacy in growth reduction in the cancer models and low impact on the control models could be confirmed. The top two drugs cladribine and gemcitabine were predicted to be effective in every cancer model and thus to be universal drug candidates, while having only minimal effect on the ATP maintenance of any control model (Fig. 4). Both gemcitabine and cladribine represent established chemotherapeutic drugs that inhibit reactions in the nucleotide interconversion pathway, responsible for DNA replication, and hence affect cell proliferation and biomass production. The 28 predicted drugs for melanoma treatment showed biomass-reducing effects also on models reconstructed for other cancer types according to TCGA datasets (Fig. 4). Thus, common alterations appear to render cancer metabolism less robust and make multiple cancer types susceptible to these drugs.

Taken together, 28 drug candidates predicted by the SKCM and IN-HOUSE consensus models were predicted to be efficient in most of the melanoma sample-level models, making these promising candidates for experimental validation.

### Cladribine, gemcitabine, lovastatin and tamoxifen have an inhibitory effect on melanoma cell lines in vitro, also in combination with conventional targeted kinase inhibitors

**Fig. 5:**
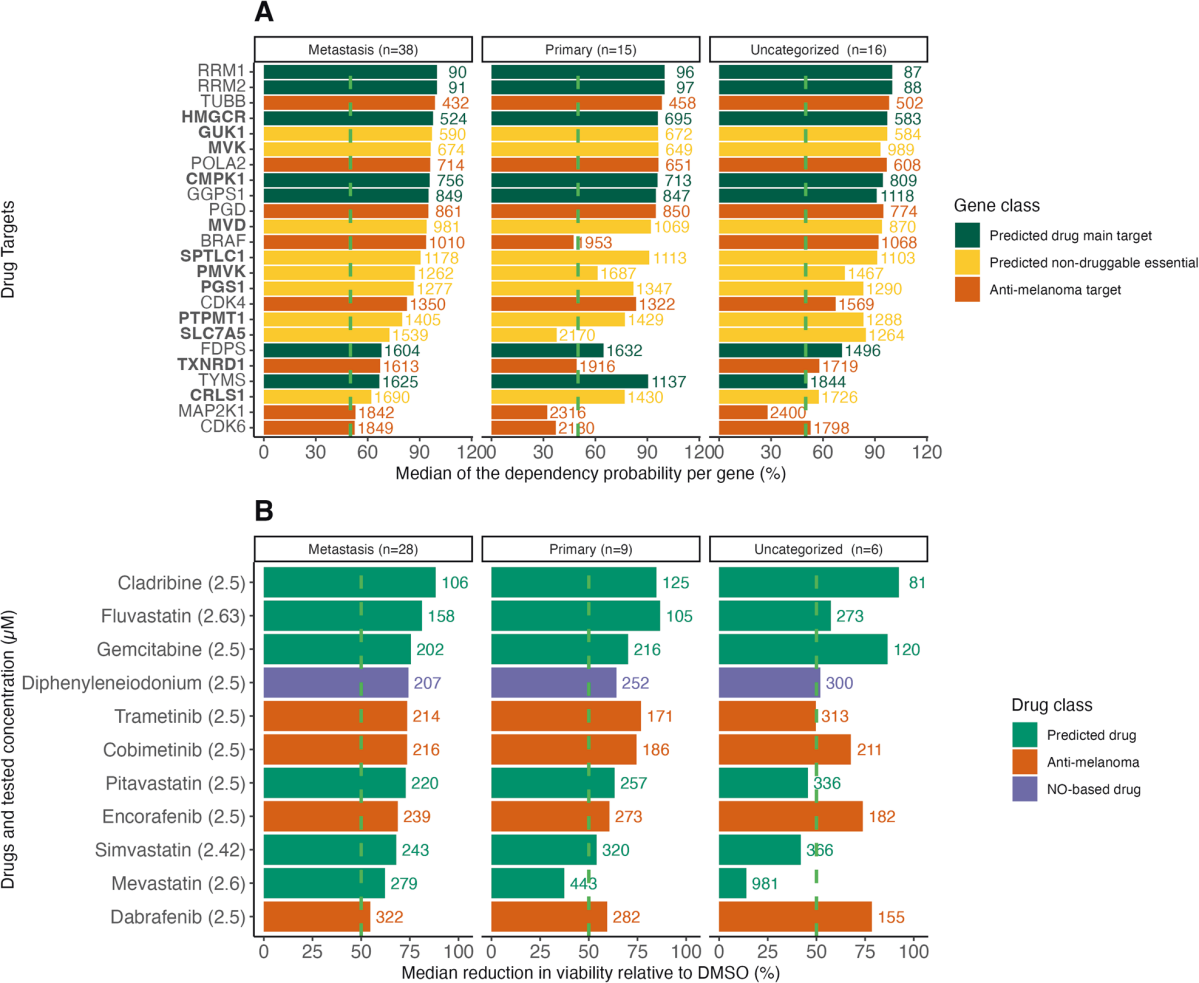
Predicted drug targets and drug candidates rank among the top metabolic candidates in the PRISM and DepMap databases. **A** Genes are ranked by their median dependency probability on the DepMap CRISPR screens [36] in the metastatic melanoma cell lines. Predicted essential genes (in bold), targets of anti-melanoma drugs (“Anti-melanoma target”) and NO-related genes were included as well. Essential genes with no predicted drugs (“Predicted non-druggable essential”) were coloured in yellow. **B** Drugs are ranked by their median reduction in viability relative to DMSO in the metastatic melanoma cell lines. Genes and drugs in **A** and **B** with a score >50% in metastatic cell lines are displayed (see Supplementary Figs. 11 and 14 for the complete drug ranking).

To identify the most promising drug candidates for in vitro testing, we filtered the predicted drugs based on their predicted efficacy, prior evidence from high-throughput CRISPR [34] and drug screens [35], IC_50_ values in melanoma and other cancer types, known metabolic targets, mechanisms and availability of clinical trial data in phase II or higher (Table 2 and Supplementary File 2). Among the predicted drugs, cladribine, fluvastatin and gemcitabine showed a stronger viability reduction in the primary PRISM database [35] than anti-melanoma and NO-based drugs in both metastatic (Supplementary Fig. 11) and treatment-resistant cell lines (Supplementary Figs. 12 and 13). Only main targets in the Drug Repurposing Hub database were considered for an in-depth discussion [36]. The main targets of the predicted drugs and our list of predicted essential genes had a higher median dependency probability (likelihood that the knock-out of a gene reduces cell growth or induces cell death) than targets of known anti-melanoma drugs (Fig. 5A and Supplementary Figs. 14 and 15). Among NO-related genes, ASS1 has the highest dependency probability (30%) and only diphenyleneiodonium induced a reduction of viability above 50% (Fig. 5B and Supplementary Fig. 14). Similarly, NO-based drugs and genes induced a low viability reduction and dependency, also in resistant cell lines (Supplementary Figs. 13 and 15).

**Table 2:** Overview of the candidate drugs selected for in vitro validation.

Drug	Approved as cancer drug	Metabolic targets (Recon 2.04)	Main metabolic targets	Non-metabolic main targets	Indication	Mode of action	Use in cancer research
Fluvastatin	No	HMGCR, CYP3A4, SLC15A1, CYP2C8, SLCO1B1, CYP2C9, CYP2C19	HMGCR	–	Hypercholesterolemia	HMG-CoA reductase Inhibitor	Anti-proliferative effects in breast cancer (Garwood et al. [76]), could prevent the onset of renal cancer (Horiguchi et al. [12]), potential synergistic effects with gemcitabine (Bocci et al. [77]) and cisplatin (Taylor-Harding et al. [78])
Ellagic Acid	No	CYP2E1, CA6, SQLE, CA12, CA3, CA9, CYP1A1, CA1, CA4, CA5B, CA5A, CA7, CA2, CA14	CA6, SQLE, CA12, CA3, CA9, CA1, CA4, CA5B, CA5A, CA7, CA2, CA14	CSNK2A1, GSK3B, PRKACA, PRKCA, PRKCB, SYK	Phytochemical abundant in fruits and vegetables	Squalene epoxidase (SQLE) inhibitor)	Apoptotic and anti-angiogenic effects in cancer cells (Losso et al. [60])
Icatibant	No	ANPEP	–	BDKRB2	Orphan drug used for hereditary angioedema treatment (Cicardi et al. [79])	Alanyl Aminopeptidase protein (ANPEP) Inhibitor	–
Terbinafine	No	CYP19A1, SQLE, CYP11A1, CYP2D6	SQLE	–	Antifungal agent	Possibly by targeting SQLE	Demonstrated anticancer effects in vitro (Chien et al. [80]; Lee et al. [81])
Tioconazole	No	CYP2E1, CYP19A1, CYP51A1, CYP3A4, CYP2C8, CYP2C19	–	–	Antifungal agent	Inhibits the ergosterol synthesis	Desensitises cancer cells to chemotherapy (P.-F. Liu et al. [65]))
Lovastatin	No	HMGCR, CYP3A4, SLCO1A2, CYP2D6, CYP2C8, SLCO1B1, CYP2C9	HMGCR	HDAC2, ITGAL, NR1I2	Hypercholesterolemia	Mevalonate pathway and cholesterol synthesis inhibitor	Anti-proliferative properties in cancers (Agarwal et al. [82]; Martirosyan et al. [83]),
Gemcitabine	Yes	TYMS, CMPK1, RRM1	TYMS, CMPK1, RRM1	–	Pyrimidine analogue	–	Inhibits DNA replication (Noble and Goa, [84]) and has been approved for the treatment of several cancers
Cladribine	Yes	RRM1, RRM2, RRM2B	RRM1, RRM2, RRM2B	POLA1, POLE, POLE2, POLE3, POLE4	Purine analogue	Ribonuclease reductase inhibitor	Used in the treatment of hairy cell leukaemia (Bryson and Sorkin, [85])
Butenafine	No	SQLE	SQLE	–	Antifungal	SQLE inhibitor	Reduces cancer proliferation (Cirmena et al. [86])
Cerulenin	No	FASN	FASN	–	Antifungal	FASN inhibitor	Induces apoptosis in human breast cancer (Liu et al. [87]; Thupari et al. [88]) and in A375 melanoma cell line (Ho et al. [89]). Suppression of colon cancer metastasis in mice liver (Murata et al. [90])
Atovaquone	No	DHODH, CYP2C9	–	–	Ubiquinone analogue used for malaria	DHODH inhibitor	Inhibits oxidative phosphorylation in cancer (Fiorillo et al. [61]), (Ashton et al. [91])
Tamoxifen	Yes	CYP19A1 CYP1B1, EBP, CYP3A4, CYP2B6, CYP2D6, CYP2C8, ABCB11, CYP2C9	EBP	ESR1, ESR2, GPER1, PRKCA, PRKCB, PRKCD, PRKCE, PRKCG, PRKCI, PRKCQ, PRKCZ	Anti-oestrogen	–	Developed to treat breast cancer (Buckley and Goa [92])

12 candidate drugs for repurposing in melanoma were selected for experimental validation. Half of the drugs are already FDA-approved as anticancer agents. Metabolic targets represent inhibited targets in the generic metabolic reconstruction Recon 2.04. The main metabolic targets in addition to non-metabolic targets were identified from the manually curated database Drug Repurposing Hub. Indication, mode of action, and use in cancer research were retrieved from Drug Bank and from literature.

**Fig. 6:**
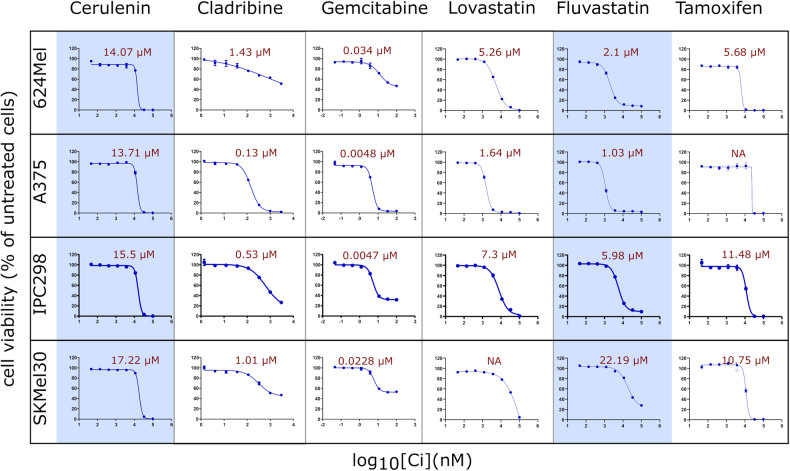
Six drugs show an inhibitory effect with a low IC_50_ values in a melanoma cell line panel. Cell viability assay at eight concentrations were performed on the 12 most promising predicted drugs. Dose response curves (representative experiment of 3) are depicted for the six most responsive drugs. IC_50_ values (indicated in red) were averaged from three experiments. Two FDA-approved drugs for non-cancer-related diseases, cerulenin and fluvastatin (blue background), showed comparable inhibiting effects on melanoma cell lines as FDA-approved anticancer (non-melanoma) drugs (white background). For lovastatin on SKMel30, the IC_50_ value could not be determined (NA).

Furthermore, tamoxifen was found to improve overall complete and partial response in combination with chemotherapies without improving 1-year survival in advanced melanoma in a meta-analysis of nine clinical trials [37]. On the contrary, melatonin (NO-based drug) combined with dacarbazine failed to show an additive effect compared to dacarbazine alone in an early terminated phase 2 trial of metastatic melanoma [38]. Interestingly, a phase 2 placebo-controlled preventive trial found that lovastatin may decrease the incidence of melanoma without reducing melanoma biomarkers [39]. Gemcitabine and itraconazole in non-melanoma skin cancer phase 2 trials show no response but stable disease in a subset of the treated patients (35% in gemcitabine [40] and 21% [41] to 91% [42] in itraconazole the disease remained stable) (see Supplementary File 2). While cladribine is still untested in a clinical trial for melanoma, it can be administered subcutaneously for cancer [43]. Using IC_50_ values and prior knowledge as criteria, we selected 12 out of the 28 predicted drugs (3 FDA-approved non-melanoma anticancer and 9 FDA-approved for other diseases) for experimental validation. Two predicted drugs (gemcitabine and cladribine) and one NO-based drug (diphenyleneiodonium) have a reported median IC_50_ below 0.4 µM in melanoma cell lines, this being comparable to known anti-melanoma drugs (Supplementary Fig. 16A). Unlike targeted anti-melanoma drugs that tend to be more effective for either BRAF-mutant or NRAS-wildtype cell lines (Supplementary Fig. 17), cladribine and other predicted drugs show good efficacy regardless of the mutation status with narrow IC_50_ ranges (see Supplementary File 2 for the IC_50_ values found in the databases and the literature and additional information on clinical trials). The selected 12 drugs were then tested in a cell viability assay on NRAS (IPC298, SKMel30) and BRAF (A375, 624Mel) mutated melanoma cell lines and the IC_50_ values for each drug and cell line were computed. Four out of the six selected drugs (cladribine, gemcitabine, lovastatin (approved for other diseases), and tamoxifen) showed an inhibitory effect on the viability of all four melanoma cell lines (Fig. 6). Fluvastatin and cerulenin, which target HMGCR and FASN, respectively, were drugs approved for non-cancer-related diseases that reduced viability of melanoma cell lines. The remaining six experimentally tested drugs did not have an inhibitory effect based on the measured dose-response curves (Supplementary Table 4).

**Fig. 7:**
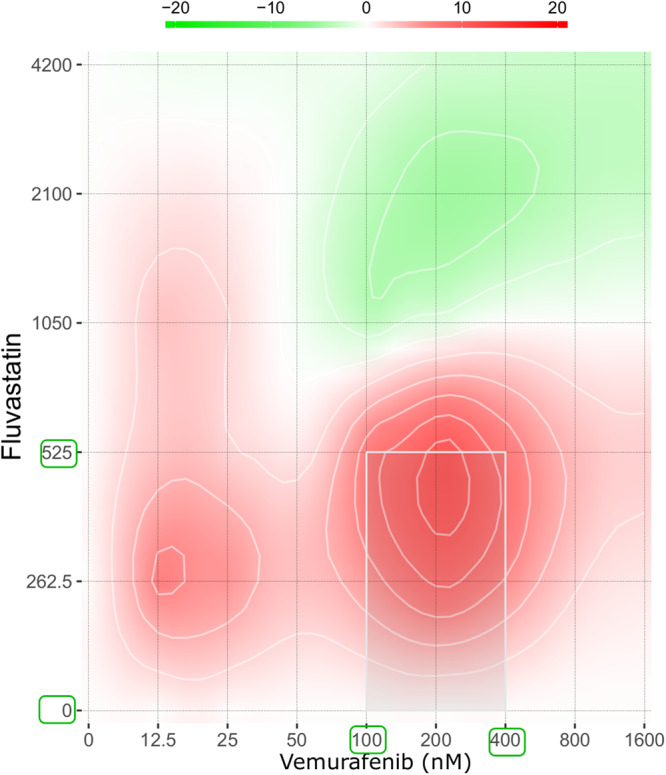
Additive and synergistic effects were observed for the combination of fluvastatin and vemurafenib in a cell viability assay for the A375 cell line. Dose-response landscape is shown as calculated by SynergyFinder and indicating Zero Interaction Potency (ZIP) scores (indicated in the colour bar, see “Methods” section). Additive/synergistic and antagonistic dose regions are represented in red (ZIP > 0) and green colours (ZIP < 0), respectively, and allow for the determination of the best concentrations for drug combination. The most synergistic area (ZIP > 10) is depicted with a grey box. The concentration bounds of the region of highest synergy are marked by green boxes on the *x* and *y*-axis.

To further examine if the six effective drugs could be beneficially combined with conventional targeted kinase inhibitors as used in the clinic, we tested the effect of gemcitabine, cladribine, fluvastatin, and lovastatin on the viability of different melanoma cell lines, respectively. Similarly, each of the four drugs were tested in combination with three BRAF inhibitors (vemurafenib, dabrafenib, encorafenib), one MEK inhibitor (binimetinib), and one selective inhibitor of the cyclin-dependent kinases CDK4 and CDK6 (palbociclib), each. The combination of BRAF inhibitors and the candidate drugs tested on BRAF-mutated cell lines yielded additive (ZIP > 0) or even synergistic effects (ZIP > 10) on cell viability, with scores between 1.8 and 15.4 (Supplementary Table 5**)**. The A375 cell line yielded ZIP scores of 13.1, 3.0 and 6.8 for the combinations of fluvastatin-vemurafenib (Fig. 7), gemcitabine-vemurafenib, and fluvastatin-encorafenib, respectively. The 624Mel cell line was slightly more responsive with ZIP scores of 15.4, 14.0, 13.4 in response to the same three drug combinations (Supplementary Table 5). For the MEK inhibitor binimetinib combinations with gemcitabine, cladribine, fluvastatin, and lovastatin, yielded mostly additive effects on cell viability of the four tested NRAS or BRAF-mutated cell lines (Supplementary Table 6). However, the CDK4/6i palbociclib acted antagonistically in all the tested cell lines (Supplementary Fig. 18), and therefore could be excluded from the panel.

**Fig. 8:**
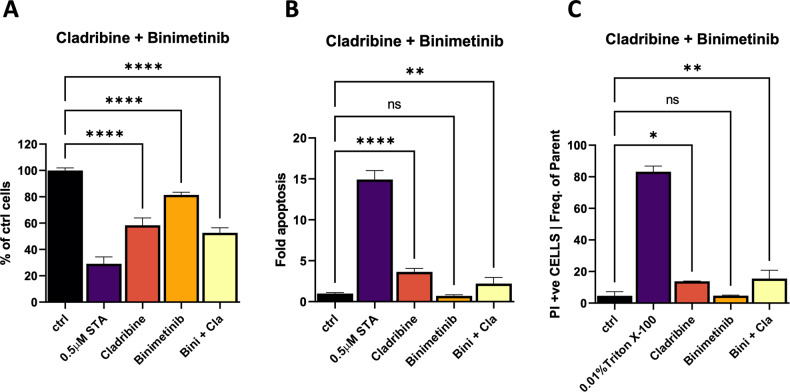
Cladribine induces cell death as single treatment. Proliferation (**A**), Propidium Iodide (PI) dead cell staining (**B**) and Caspase 3/7 Ac-DEVD-AFC apoptosis assays (**C**) of cladribine and binimetinib are shown for the 624Mel cell line. Assays were performed as detailed in Supplementary File 1. ANOVA analysis was performed on *n* = 2 replicates. Statistical significance is indicated as: ^ns^*p* ≥ 0.05, *p < 0.05, **p < 0.01, ***p < 0.001, ****p < 0.0001.

Furthermore, cell death and proliferation assays were performed for these four drugs (cladribine, gemcitabine, fluvastatin, and lovastatin) in mono- and combination therapy with targeted inhibitors in two cell lines (624Mel, BRAF mutated and SKMel30, NRAS mutated). Cladribine decreased proliferation and increased the PI dead cell count, while inducing apoptosis (Fig. 8; Supplementary Fig. 21). Whereas gemcitabine also showed some promising results in SKMel30 cells, the other two drugs, fluvastatin and lovastatin, as well as the combination with palbociclib (CDK4/6i), binimetinib (MEKi) or vemurafenib (BRAFi) did not further increase apoptosis or the PI dead cell count (Supplementary Figs. 19–27). However, the proliferation assays showed a significant decrease in proliferation for all candidate drugs when combined with binimetinib (MEKi; Supplementary Fig. 19).

Taken together, we have identified four drugs that show an additive/synergistic inhibitory effect in the proliferation assay and as monotherapy in the viability screen in all four tested melanoma cell lines. These drugs should be considered to be used as an extension to the panel of conventional drugs for combined melanoma treatment.

## Discussion

In this study, we used metabolic network modelling to identify drug targets and candidate drugs for alternative melanoma treatment to be used individually or in combination with conventional targeted kinase inhibition. Most of the drugs identified by applying our workflow could potentially act as pan-cancer drugs, which target de novo nucleotide and fatty acids synthesis pathways as well as oxidative phosphorylation.

Cancer cells often rely on pathways that are under tight regulation in healthy tissues, including the de novo cholesterol, lipid, and nucleotide synthesis pathways [25, 44, 45] to increase tumour mass and sustain high proliferation [46]. Some of the predicted drug targets by our refined workflow, namely HMGCR, FASN and SQLE are key regulators of cholesterol and lipid homeostasis. These genes were shown to be overexpressed in several cancers [47] favouring cell migration and proliferation [48]. FASN is also an unfavourable prognostic marker for various cancers including melanoma [49] and high expression values for this gene correlates with advanced stages of colon, breast, and prostate cancer [50]. Besides contributing to the increase of tumour mass, the rewiring of cholesterol and sphingolipids metabolism alters the composition of lipid rafts that play an important role in other hallmarks of cancer such as oncogenic signalling, migration, proliferation, adhesion, invasiveness, metastatic spread, apoptosis evasion [51], vesicular trafficking and drug resistance [52]. Furthermore, an imbalance in cholesterol and sphingolipids metabolism allows cancer cells to proliferate, to escape the immune system [53] and to invade other tissues [54]. Hence our predicted drugs could allow targeting several hallmarks of cancer simultaneously.

Cancer cells have high energy demands. Although fast-cycling cells mostly rely on the Warburg effect, a minority of cancer cells still prefers OXPHOS, especially in advanced stages and upon acquired drug resistance [55]. Accordingly, genes implicated in and regulating OXPHOS, like PISD and PKM2, were also predicted as potential drug targets in our model analysis. In general, the efficacy of a drug to fight metastatic cells might depend on the preferred source of energy in concert with the proliferation efficacy. Drugs that reduce the proliferation of fast-cycling cells might be less effective in migrating cells, which often rely on OXPHOS and hence are not as dependent on de novo synthesis pathways and DNA replication. Current therapies based on MEK, BRAF and CDK4/6 inhibitors tend to induce resistance by activating OXPHOS and fatty acid oxidation, thus reducing the tumour mass but increasing the risk of relapse [56].

Most drug targets identified using our drug deletion pipeline overlap with the predicted essential genes like SQLE, FASN and HMGCR or are members of the same pathways. However, in some cases drugs are only predicted to be effective if multiple of their targets are knocked out in the drug deletion pipeline. This is notably the case for CYP proteins and the Carbonic Anhydrase (CA) family of zinc metalloenzymes. CYPs play a role in tumour initiation, drug activation and clearance [57]. CAs were shown to promote tumorigenesis by maintaining the pH in a favourable range for the tumours and promote metastasis by reducing cell adhesion [58].

In the experimental validation, we have shown that six out of the 12 selected drugs had an inhibitory effect on melanoma cell viability. For the remaining six drugs literature evidence supports their predicted efficacy. Three drugs (butenafine, terbinafine, ellagic acid) did not show an effect, despite their drug target (SQLE) being considered a promising target in cancer therapy [59]. Ellagic acid showed anticancer properties by decreasing the levels of ATP within different cancer cells [60] and was registered in a clinical trial for dietary intervention in follicular lymphoma (NCT00455416). It might still show an inhibitory effect at higher concentrations. The other three drugs which failed in the experimental validation were atovaquone, icatibant and tioconazole, but showed promising results in other studies: atovaquone was previously shown to inhibit cellular respiration in breast cancer cells at a concentration of 5 µM [61], to increase oxygenation of tumours [62] and platinum-mediated cell death due to oxidative stress. Icatibant can be used to prevent the accumulation of fluids in the peritoneal cavity of patients suffering from ovarian cancer [63, 64] and tioconazole was shown to increase the cytotoxic effects of doxorubicin [65]. Also, one of the metabolic targets of icatibant is ANPEP, which is a prognostic marker for prostate cancer [33]. Finally, FASN has long been a promising target for anticancer therapy, but so far no drug has proceeded to the clinics [66]. The predicted drug cerulenin targeting FASN showed in our screen IC_50_ values above the recommended therapeutic plasma concentration of 10 µM and it has therefore not been considered for further synergy testing. Cladribine, gemcitabine, lovastatin, and tamoxifen overall have lower IC_50_ than the NO-based drugs, which are part of a few cancer and melanoma clinical trials (NCT00060710, NCT05502900) and pronouncedly reduced viability in both, metastatic and therapy-resistant melanoma cell lines (see Supplementary File 1).

These four compounds additive and partially synergistic effects with BRAFi or MEKi. For example, gemcitabine showed promising results in combination with the MEKi binimetinib in NRAS mutant cells. However, the effects of the individual drugs and the combinations applied appeared to have cytostatic rather than cytotoxic effects. Fluvastatin and lovastatin target the cholesterol pathway that plays a role in the prenylation of members of the PI3K/AKT/mTOR pathway and RAS, which could impact on the ability of RAS to activate BRAF [67], eventually causing a proliferation arrest. Cladribine is an antimetabolite that causes cell arrest in G1 in B-cell lymphoma cells by modulating the activity of apoptotic proteins, notably c-Flip_L,_ Bax, and Death receptor 4 (DR4) and Caspase 8. Cladribine furthermore activates endoplasmic reticulum stress, further inducing apoptosis [68]. Gemcitabine induces cell-cycle arrest concomitant with caspase-3-mediated apoptosis [69]. Interestingly, gemcitabine has already been proposed as an adjuvant in combination with another MEKi, trametinib, in adenocarcinoma treatment, but due to low efficacy, the studies were terminated [70]. A recent study showed that gemcitabine combined with cobimetinib might effectively treat KRAS-mutated pancreatic cancer [71]. While gemcitabine was found inactive in a phase 2 trial against non-melanoma skin cancer with 35% stable disease and short 6 months median overall survival, it is still to be investigated in melanoma [40]. Fluvastatin, in turn, presented with high synergy scores in combination with three tested BRAFi, whereas cladribine showed effectiveness in combination with both, BRAFi and MEKi. Concerning statins, studies have demonstrated their anti-tumour effects in several cancer [15, 72] including on melanoma as single drug [13, 16] or in combination with cisplatin [73]. In addition, a recent study identified cladribine as a possible repurposable drug in CDKN2A mutated melanoma using data mining followed by in vitro validation [74]. Hence, cladribine showed efficacy with narrow IC_50_ regardless of the melanoma cell lines' resistance, metastasis or mutation status, making it a promising candidate for melanoma clinical trials.

Taken together, our study demonstrates how in silico drug target prediction based on metabolic modelling can be a useful complement in the selection of tailored treatments to improve the therapeutic outcome of melanoma patients especially for those that relapse or do not respond to current melanoma treatments. The low computational demands and the robustness of rFASTCORMICS allowed the reconstruction of thousands of sample models that allow assessing how many patients share a metabolic alteration that can be exploited as drug target and hence allow us to identify metabolic rewiring strategies that are common across cancer patients and more particularly across melanoma patients.

## Supplementary information

Supplementary File 1

Supplementary File 2

aj-checklist

confirmCoauthorAliKishk

## Data Availability

The consensus models and rFASTCORMICS can be downloaded from https://github.com/sysbiolux/MelanomaPaper and https://github.com/sysbiolux/rFASTCORMICS. Raw FASTQ files were submitted to the European Genome-phenome Archive (EGA) database, accession number: EGAS00001006463. The FPKM can be obtained from the authors on request.
