# Supplementary material for: Metabolic modelling-based in silico drug target prediction identifies six novel repurposable drugs for melanoma
Source: Cell Death Dis. 2023 Jul 26;14(7):468. doi: 10.1038/s41419-023-05955-1 (PMC10372000; doi:10.1038/s41419-023-05955-1)
Supplement: Supplementary file 1 [file 41419_2023_5955_MOESM1_ESM.pdf]

In silico drug target prediction using metabolic modelling identifies six novel repurposable drugs for melanoma - Supplementary File 1

Tamara Bintener[1#], Maria Pires Pacheco[1#], Demetra Philippidou[1], Christiane Margue[1], Ali Kishk[1], Greta Del Mistro[2,3], Luca Di Leo[5], Maria Moscardó Garcia[1], Rashi Halder[4], Lasse Sinkkonen[1], Daniela De Zio[5], Stephanie Kreis[1], Dagmar Kulms[2,3], Thomas Sauter[1*]

Corresponding Author: Thomas Sauter, Department of Life Sciences and Medicine, University of Luxembourg, Esch-Alzette, Luxembourg, Thomas.Sauter@uni.lu

# These authors contributed equally.

Supplementary Materials and Methods

0.1 Cell culture (IN-HOUSE dataset and validation)

Patient brain and systemic metastasis (TUMEL32/32H) samples were kindly provided by Drs Meier and Niessner, University of Tübingen, Germany. Melanocytes were purchased from Cell Systems (Troisdorf, Germany) and used at passage 4 in Melanocyte Growth Medium (M2, Promocell, Heidelberg, Germany). The IN-HOUSE cell lines were acquired from the American Type Culture Collection (ATCC). MALME 3M, WM1366, WM134, A375 and A375 desensitised to IZI (A375IZI) were cultivated in RPMI 1640 medium (Invitrogen, Karlsruhe, Germany) while MeWo and SK-Mel-5 were grown in Advanced Minimum Essential Media (MEM) (ThermoFisher Scientific, MA, USA; cat# 12492-021) supplemented with 2 mM GlutaMAX™ (ThermoFisher Scientific; cat# 35050-038). All culture media were supplemented with 10% FBS (ThermoFisher Scientific; cat# 10270-106) and 100 U/ml P/s, maintained in an atmosphere of 5% CO2 in air at 37°C and passaged no more than 18 times before a low-passage cryovial was thawed and routinely tested for Mycoplasma contamination (eurofins Genomics, DE). All but 6 samples were single-end sequenced at the Luxembourg Centre for Systems Biomedicine. The other samples (3xMW and 3xSK5) were paired-end sequenced by BGI Europe A/S (Copenhagen, DK). After the cleaning and filtering, the reads were mapped to the reference genome. For the validation, the BRAF-mutant melanoma cell line A375 was purchased from ATCC, while 624Mel cells were obtained from Dr Halaban (Yale School of Medicine, USA). The NRAS-mutant melanoma cell lines SKMel30 and IPC298 were obtained from DSMZ Germany. These cells were maintained, as described above in RPMI-1640 containing GlutaMAX™ (GIBCO) and supplemented with 10% FCS (GIBCO), 100U/ml penicillin and 100μg/ml streptomycin (GIBCO).

0.2 RNA-seq analysis

Total RNA from the 31 samples (3xA375, 4xA375-IZI, 3xMalme3M, 3xMold, 3xTumel323, 3xTumel32H, 3xWM1346 and 3xWM1366) were single-end sequenced at the Luxembourg Center for Systems Biomedicine. The other 6 samples (3xMW and 3xSK5) were paired-end sequenced by BGI Europe A/S (Copenhagen, DK. RNA sequencing was performed on RNA extracts from SK-Mel-5 and MeWo cell lines (500 ng) by BGI Europe A/S (Copenhagen, DK). Samples were sequenced with DNBseq platform averagely generating about 8.31 Gb bases per sample. The raw FASTQ were trimmed by Adapter Removal (Schubert et al., 2016) which removed both read 3' end of poor quality (quality score of ≤ 2) and also match to the Illumina adapter sequence AGATCGGAAGAGCACACGTCTGAACTCCAGTCAC. and the pipeline workflow PALEOMIX (Schubert et al., 2014). The samples sequenced at the LCSB were processed using the human genome version GRCh38.p1, with the GENCODE annotation v20 and indexed by the splice-aware align STAR (v2.5.2b (Dobin et al., 2013) using –sjdbOverhang 74). Trimmed reads were then mapped against the indexed human genome using the following tweaks:

$--limitOutSJcollapsed$ 1000000 $--limitSjdbInsertNsj$ 1000000

$--outFilterMultimapNmax$ 100 $--outFilterMismatchNmax$ 33

$--outFilterMismatchNoverLmax$ 0.3 $--seedSearchStartLmax$ 12

$--alignSJoverhangMin$ 15 $--alignEndsTypeLocal$

$--outFilterMatchNminOverLread$ 0 $--outFilterScoreMinOverLread$0.3

$--winAnchorMultimapNmax$ 50 $--alignSJDBoverhangMin$ 3

As expected, most reads were uniquely aligned (LCSB: 85.1-93.6%, ranging from 21.63 - 50.71 million reads; BGI: 97.1-97.3%, 40.0-40.5 million reads).

BAM files served as input for featureCounts from the R package Rsubread (Liao et al., 2019) and resulting matrix of counts summarised at the gene level loaded in the bioconductor R package DESeq2 (Love et al., 2014). After adding the gene sizes to the DESeq2 object, FPKM were obtained with the function fpkm().

For the sample sequenced by BGI Europe, the filtering and clean reads were mapped to reference genome using HISAT2 (Kim et al., 2015). Gene expression level for each sample was quantified with RSEM (Li and Dewey, 2011).

0.3 Drug prioritisation

The effect of a knock-out of the essential genes, predicted targets, main targets of anti-melanoma drugs, and genes implicated in NO metabolism (NO-related genes) retrieved from the Drug Repurposing Hub (Corsello et al., 2017) and literature (Mintz et al., 2021), (Stasch et al., 2011), were compared based on the median dependency probabilities (the likelihood that the knock-out of a gene reduces cell growth or induce cell death) in the CRISPR-Cas9 Cancer Dependency Map (DepMap) database (Pacini et al., 2021) across melanoma primary, metastatic and uncategorised melanoma cell lines. Likewise, drug-induced resistance melanoma cell lines were used to rank these drug targets and essential genes. The resistance information was extracted from the "depmap_public_comments" column in the cell line metadata.

The viability reduction against DMSO for predicted, anti-melanoma and NO-based drugs were assessed using the Primary PRISM database (Corsello et al., 2020) for primary and metastatic cell lines and resistant versus sensitive. Resistant cell lines were defined as cells with at least 50% proliferation to anti-melanoma drugs (-50% viability reduction). The anti-melanoma drugs consisted of nine approved anti-melanoma in the Drug Repurposing Hub (Corsello et al., 2017) in addition to the CDK4/6-inhibitor palbociclib that showed modest efficacy in a phase 2 melanoma trial (Louveau et al., 2021). The NO-based drugs were retrieved from different sources (Corsello et al., 2017), (Mintz et al., 2021), (Stasch et al., 2011), (Janakiram and Rao, 2012) and covered four mode-of-actions (see Supplementary File 2).

While the primary PRISM database covers a wide range of tested drugs (4606), these drugs were tested using a narrow range of concentrations (2.5:5 μM). On the other hand, cell

viability databases with more tested concentrations, allow determining more precise potency (such as IC_{50}) but on a smaller drug set. The three sets of drugs (predicted, NO-based, and anti-melanoma) were compared using the IC_{50} measures in high-throughput pan-cancer cell viability databases. Four databases with IC_{50} values were merged: Secondary PRISM (19Q4) (Corsello et al., 2020), GDSC1000 (Release 8.4) (Yang et al., 2013), GDSC2000 (Release 8.4) (Yang et al., 2013), and Genentech Cell Line Screening Initiative (Haverty et al., 2016). As fotemustine was the only anti-melanoma missing in the merged IC_{50} database, fotemustine IC_{50} in melanoma cell lines were added manually from the literature. The melanoma cell lines in the secondary database were further classified based on the BRAF and NRAS mutation status to identify sensitive candidate drugs regardless of the cell line mutation profile. Mutation status retrieved from the DepMap website ("Binary Calls for Copy Number and Mutation Data") was used to classify the melanoma cell lines in the secondary PRISM database based on BRAF and NRAS mutations.

0.4 Experimental validation

0.4.1 Cell viability assay

Inhibitors: All inhibitors were purchased either from Selleckchem (fluvastatin, icatibant, tamoxifen, tioconazole, vemurafenib (PLX4032), dabrafenib, encorafenib, binimetinib and palbociclib) or MedChemExpress (atovaquone, butenafine, cerulenin, cladribine, ellagic acid, gemcitabine, lovastatin, terbinafine). The compounds were dissolved in DMSO to a concentration of 10 mM and stored at -80°C.

Dose-response curves and determination of IC_{50} values: Melanoma cells were seeded at a density of 0.5×10 cells/well in 96-well black μclear plates (Greiner). 3-fold dilution series of each drug (ranging from 0.05 to 100000nM, depending on the drug) were assayed in technical triplicates for 72h (Margue et al., 2019).

0.4.2 Proliferation assay

Briefly, 624mel and SKMel30 cells were seeded at a density of 5000 cells/well in a 96-well μclear plate (655090, Greiner). Cells were treated with either the drugs alone or combinations thereof for 72h at concentrations shown in Supplementary Table S 1. At the end of the treatment, cell viability was measured on Cytation 5 (Biotek) with the PrestoblueTM Cell Viability reagent (A13262, ThermoFisher Scientific) according to the manufacturer's protocol.

0.4.3 Propidium Iodide dead cell staining

624mel and SKMel30 cells were seeded at a density of 105 cells/well in 2ml RPMI in 6-well plates (657160, Greiner). Cells were treated for 72h with the respective allocated drug amounts as shown in Supplementary Table S 1. At the end of the treatment cells were collected (supernatant, PBS wash and trypsinised cells) and stained with 1ug/ml Propidium Iodide (537059, Merck Life Sciences BV) for 15min. Positive control cells were treated with 0.01% Triton X-100 (T8787, Merck Life Sciences BV) during staining. After PBS washing, stained cells were analysed on FACSCanto II (BD Biosciences). Results were analysed with the FlowJo software (v.10.8.1, BD Biosciences).

0.4.4 Caspase 3/7 Ac-DEVD-AFC apoptosis assay

624mel and SKMel30 cells were seeded at a density of 5000 cells/well in a 96-well μclear plate (655090, Greiner). Cells were treated either with the single drugs or their predefined combinations at the concentrations shown in Supplementary Table S 1. 72h after treatment start, cells were lysed with 3x ReLy buffer (150mM Tris, pH 7.4 / 300mM NaCl / 30% glycerol, 1%Triton X-100 / 0.3% Chaps / 6mM EDTA) containing 75 μM Ac-DEVD-AFC (sc-311274A, Santa Cruz Biotechnology, Inc.). Signal specificity was checked with addition of Ac-DEVD-CHO caspase inhibitor (13403, ImTec Diagnostics N.V.) to selected wells. Treatment with 0.5 μM Staurosporine (81590, Cayman Chemicals) served as positive apoptosis control. Fluorescence intensity was measured on Cytation 5 (Biotek).

Table 1: **Drug concentrations used in proliferation, apoptosis and PI dead cell staining assays.**

A

624mel	Conc. (nM)	Combinations
PLX4032	45	
PLX4032	175	
PLX4032	400	
Cladribine	400	PLX45 + Cla400
Gemcitabine	9	PLX45 + Gem9
Fluvastatin	255	PLX175 + Fluva255
Lovastatin	665	PLX400 + Lova665

B

624mel	Conc. (nM)	Combinations
Binimetinib	12	
Binimetinib	38	
Cladribine	360	Bini12 + Cla360
Gemcitabine	5	Bini12 + Gem5
Fluvastatin	520	Bini12 + Fluva520
Lovastatin	750	Bini38 + Lova665

C

624mel	Conc. (nM)
Palbociclib	1000
Cladribine	1400
Gemcitabine	35
Fluvastatin	2000
Lovastatin	5200

SKMel30	Conc. (nM)
Palbociclib	1000
Cladribine	1000
Gemcitabine	25
Fluvastatin	22000

E

SKMel30	Conc. (nM)
Binimetinib	6
Cladribine	200
Gemcitabine	5
Fluvastatin	5000

Quality Control concerning the essential genes predictions

Models capture metabolic variations between conditions

Models of the same tissue or condition are expected to cluster together as sharing a higher similarity (high quality). If the quality of the models is low, these are not able to capture metabolic variation among the conditions. Hence, to assess the quality of the context-specific reconstruction process, the sample were clustered based on the Jaccard Similarity Index. Overall, models separated mainly along with their tissue of origin and then in function of their status (cancer or control), as previously described for the TCGA dataset (Pacheco et al., 2019), showing that the models were able to capture metabolic variations between the different tissues and also between healthy and cancer models. For the CCLE models, no clear separation in clusters was observed, as all models are derived from melanoma cell lines and do not include replicates (Supplementary Figure 1). However, for the IN-HOUSE melanoma models, the replicates in the sample models clustered together and in the consensus model, three major clusters composed of the control melanocytes, the A375 cell lines a low metastatic melanoma cell line), together with the metastatic samples, and the remaining cell lines could be observed. The third cluster could further be separated for the brain metastasis, which formed a group with WM1346 and WM1366 and another composed of MeWO, SKMel5, Malme3M. Furthermore, the A375 cell line shared metabolic similarities with both the metastatic samples and to some extent with the control melanocytes (Supplementary Figure 2).

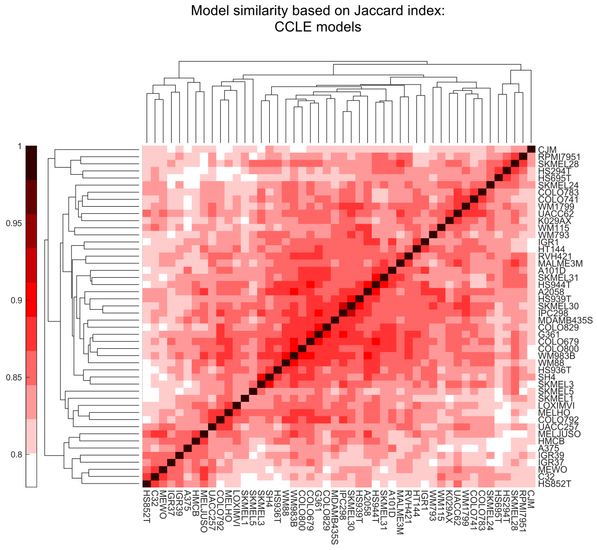

Figure S 1: **CCLE similarity.** For the CCLE dataset, no apparent cluster can be observed. The similarity between the different models is greater than 75%.

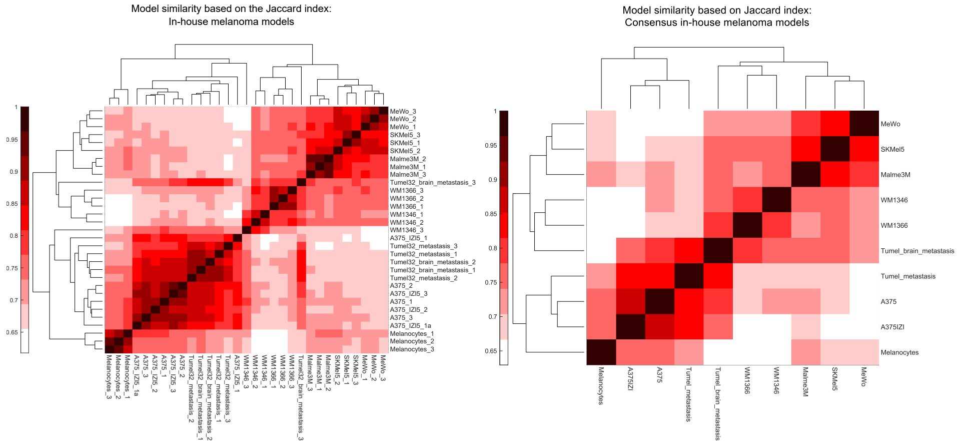

Figure S 2: **Melanoma model similarity.** For each model pair in the sample-specific and consensus selection, the Jaccard similarity score was determined to assess how similar the models are, based on the reaction presence. The data is then represented in a clustergram that allows finding clusters of similar models. For the melanoma models, three clusters can be observed for the sample-specific and consensus models. One cluster comprises the control melanocytes, the second cluster includes mainly the patient-derived Tumel models and the A375 cell line with the IZI treated, and the third cluster is formed by the remaining cell lines.

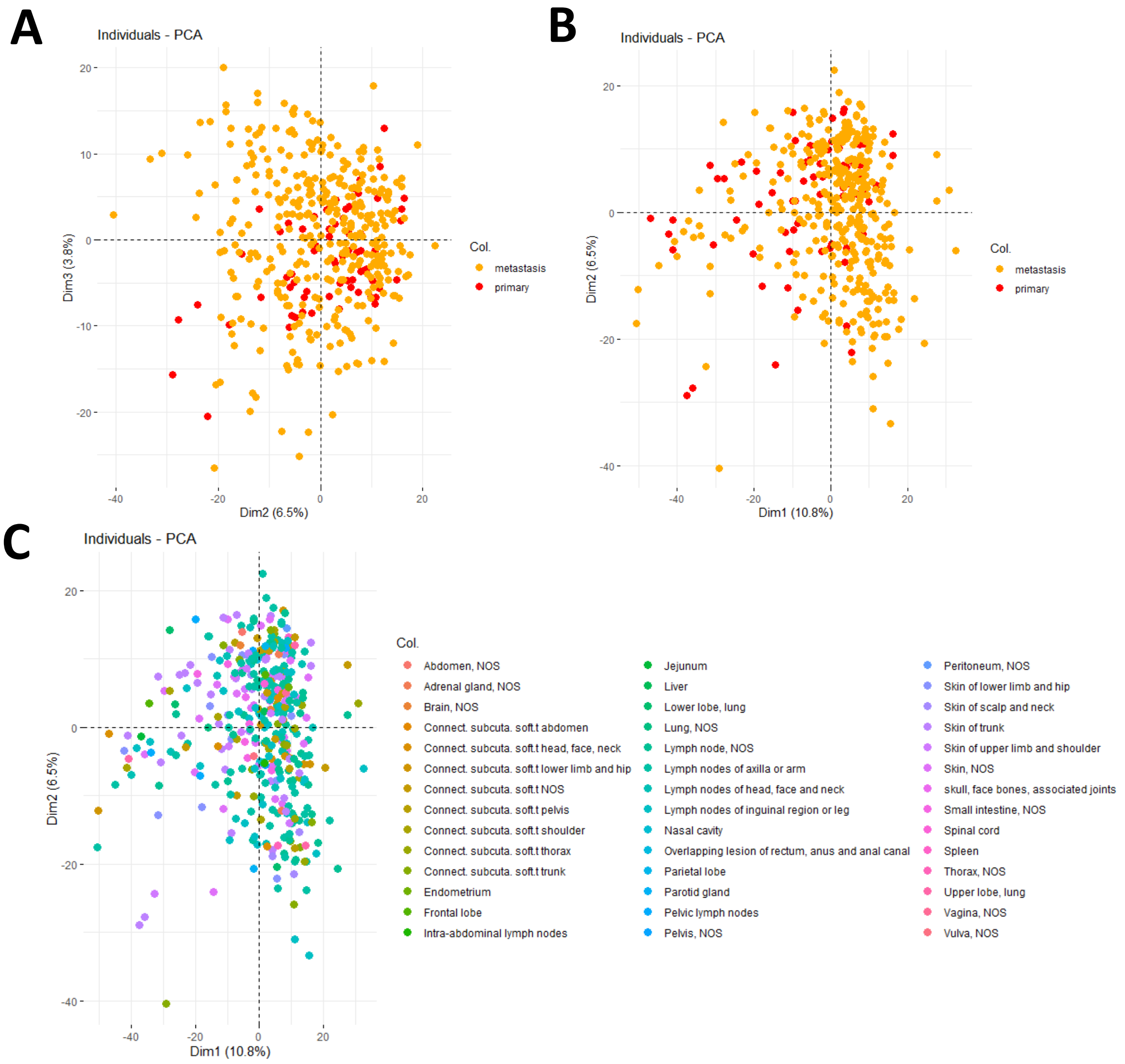

Figure S 3: **The melanoma patient (SKCM) and cell line CCLE expression data did not allow to separate primary and metastatic samples.** A PCA analysis was performed on the SKCM expression data. The 1st and 2nd dimensions (A) and the 2nd and 3rd dimensions (B) were plotted and colourised according to the stage and according to the biopsy site (C).

30 essential genes are common to TGCA, SKCM, INHOUSE models

30 essential genes are common to TGCA, SKCM, IN-HOUSE models but the knockout of these genes does no affect ATP maintenance in the liver and kidney models.

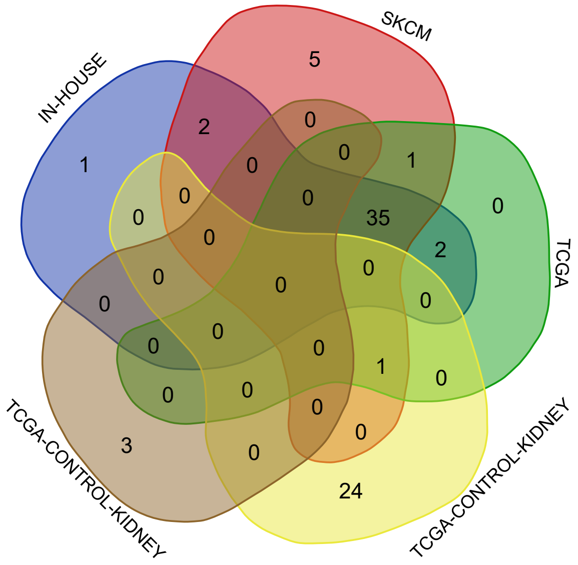

Figure S 4: **Conserved essential genes across the different melanoma, cancer cell types and healthy tissues.** Venn diagram presenting the overlaps of predicted essential genes between the consensus IN-HOUSE models, the TCGA, the SKCM, TCGA CONTROL LIVER and TCGA CONTROL KIDNEY.

Table 2: **35 genes were common to all TCGA, SKCM, IN-HOUSE models and 2 genes were specific to IN-HOUSE and SKCM**.

Essential genes	Pathways	essential
PTPMT1<br>PGS1	Glycerophospholipid metabolism	lethal when knock-out in TCGA, SKCM, IN-HOUSE model
CRLS1	Cardiolipin synthesis and Glycerophospholipid metabolism	
SGMS1<br>SPTLC1<br>SPTLC2<br>SPTLC3<br>KDSR	Spingolipid metabolism	
CMPK1<br>TXNRD1<br>CAD<br>DHODH<br>UMPS<br>GUK1	*de novo* synthesis of nucleotides	
FDFT1<br>SQLE<br>LSS<br>CYP51A1<br>MSMO1<br>EBP<br>TM7SF2<br>DHCR7<br>HMGCR<br>MVK<br>MVD<br>PMVK<br>NSDHL	cholesterol metabolism	essential when knock-out in TCGA, SKCM, IN-HOUSE model
ACACA<br>LCAT<br>LIPA<br>FASN<br>HSD17B4	*de novo* synthesis and metabolism of fatty acids	
ANPEP<br>SLC27A4<br>SLC7A5	miscellaneous	
PISD<br>PKM2		essential in IN-HOUSE and SKCM consensus model

Common versus context-specific fitness essential genes

How common or how specific a gene is, was estimated by counting the number of the sample models that predicted this gene to be essential. Common melanoma or cancer genes, if druggable, might be interesting drug targets as are likely to have a low NNT. In the main text, the analysis was shown for the 40 predicted essential genes by IN-HOUSE consensus melanoma models. Supplementary Figure 7 displays the same analysis for the top essential genes in the IN-HOUSE sample models. The same analysis was performed on the TCGA dataset (Supplementary Figure 5) to assess if a gene is a common essential gene, but also on SKCM and CCLE sample models (Supplementary Figure 6).

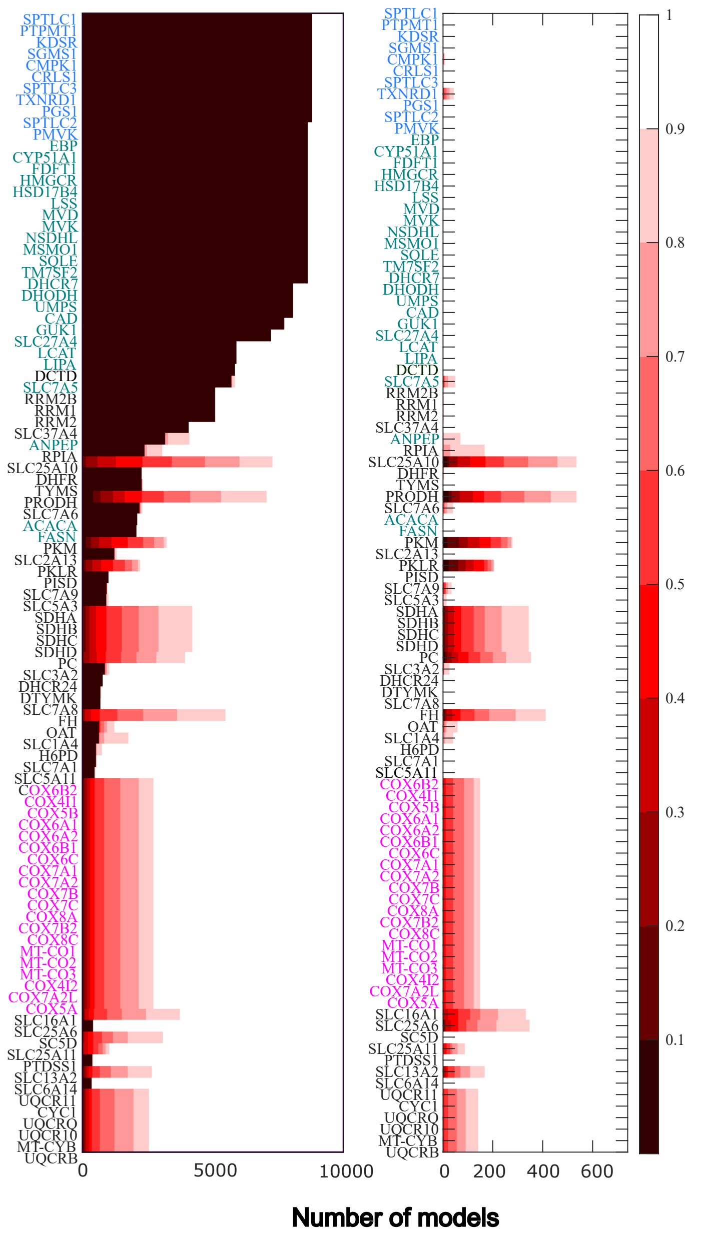

Figure S 5: **Top 100 most conserved essential in the TCGA sample models.**
In silico gene deletion analysis that shows (in x-axis) the number of models that are affected by the deleted gene. The y-axis represents the essential genes, sorted by growth ratio in the TCGA models. The growth ratio is presented in the colour scale from dark (complete reduction of the objective function) to white (no effect on the objective function).
The deletion of a set of genes (blue band) completely shuts down the biomass reaction. The deletion of the gene set within the green band only shut down biomass in some models. In the purple band are genes that are implicated in oxidative phosphorylation. The genes in the green, blue and purple bands are mostly conserved across the TCGA, SKCM, CCLE models. The arrows show the essentially for some genes of interest.

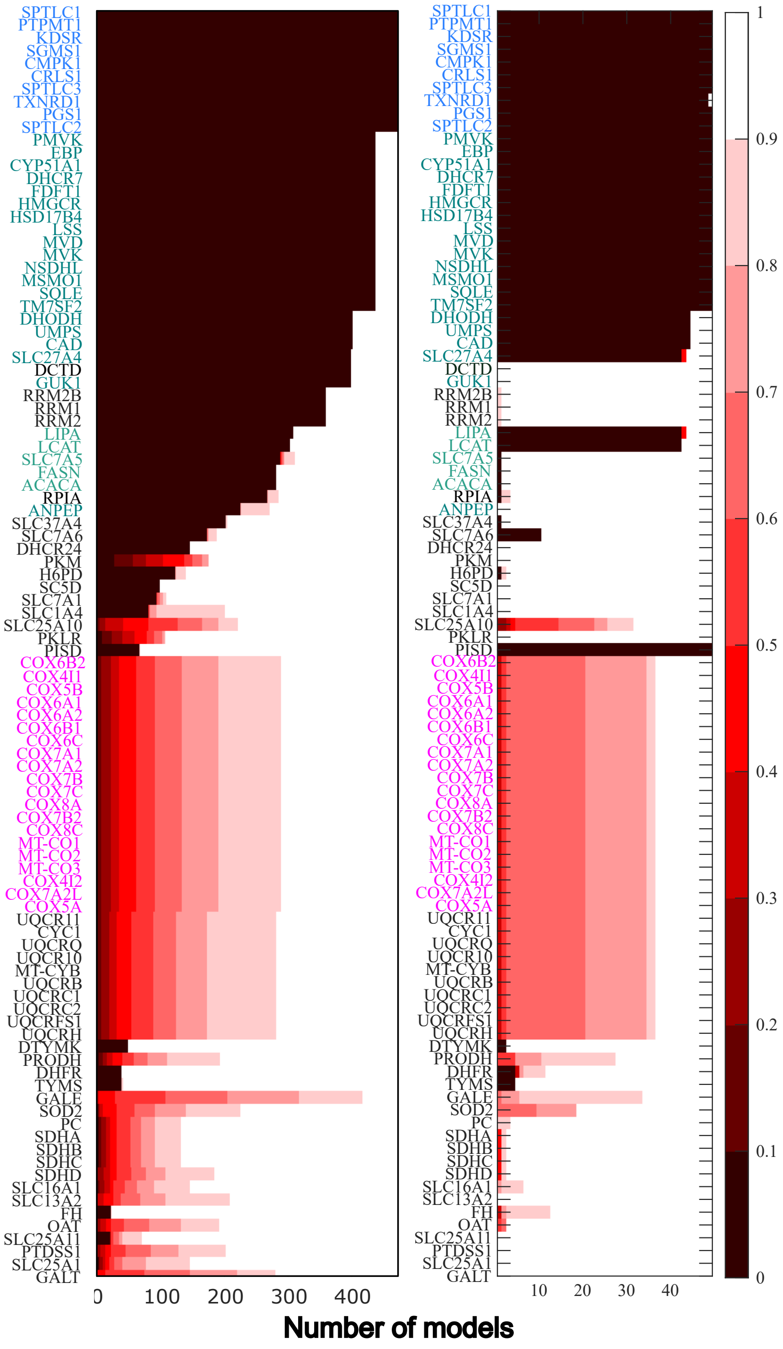

Figure S 6: **Top 100 most conserved essential in the SKCM (left) and CCLE (right) sample models.** The deletion of a set of genes (blue band) completely shuts down the biomass reaction on both SKCM and CCLE models.

The deletion of the gene set within the green band only shut down biomass in some models. In the purple band are genes that are implicated in oxidative phosphorylation. The genes in the green, blue and purple bands are mostly conserved across the TCGA, SKCM, CCLE models. The arrows show the essentially for some genes of interest.

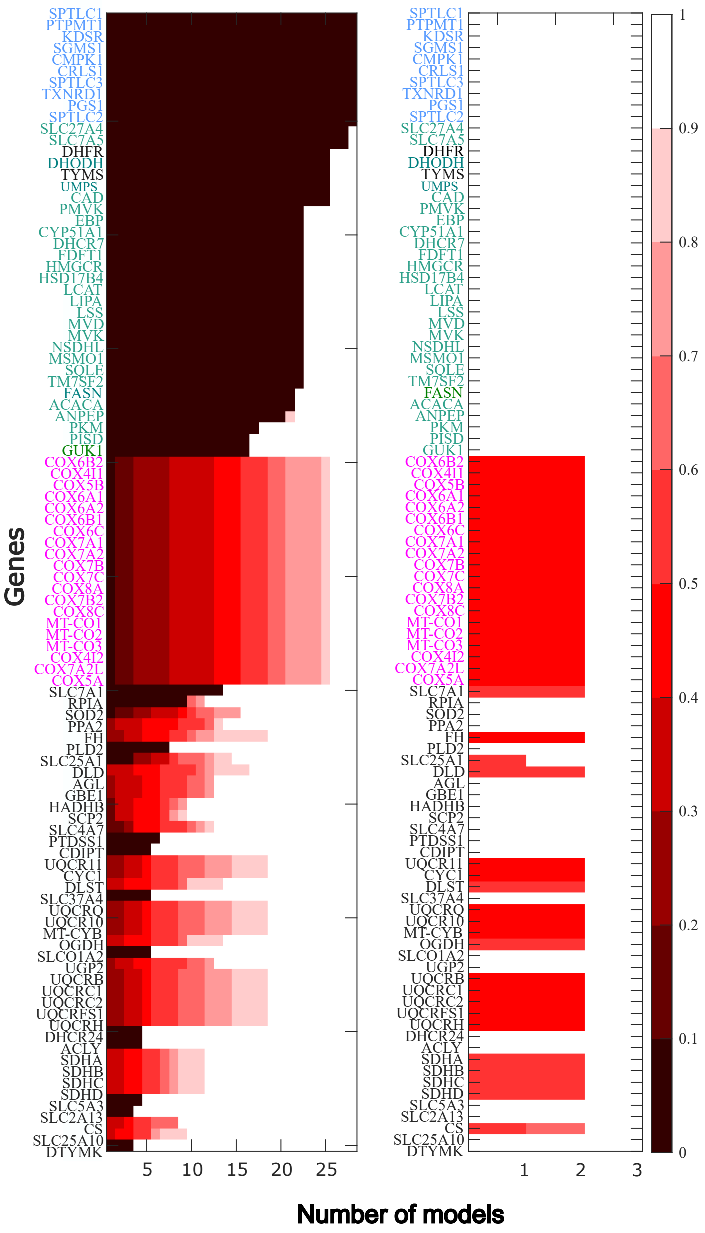

Figure S 7: **Conserved essential genes across the IN-HOUSE models.** The upper part of the left panel includes genes whose deletion completely reduces the biomass reaction to 0 in all of the 32 cancer models of the IN-HOUSE melanoma dataset (blue band). Below are sets of genes that reduce the biomass reaction to 0 in most cancer models but not all (green band). Differential effects can be observed for some genes (purple and turquoise bands) that affect only a few cancer samples and to different degrees. Some sets of genes that reduce the biomass production in the cancer models also reduce the ATP production in the control models (purple band). The arrows show the essentially for some genes of interest.

Quality control concerning the drug candidate predictions

The drug predictions of the sample models mostly overlap with the consensus models

28 shared drugs were predicted by the IN-HOUSE and SKCM consensus models. Whereas 25 were predicted by the sample models across the 4 datasets. (Supplementary Figure 8). To further access the robustness of the prediction, the consensus models were compared to the sample models. The same 41 drugs were predicted by the two types of IN-HOUSE melanoma models (consensus and sample models), whereas for SKCM models 80 drugs were predicted by the consensus against 31 for sample suggesting a higher heterogeneity in the SKCM dataset. All the models shared at least 25 drugs further confirming the robustness of the predictions (Supplementary Figure 9). The same analysis was performed among the cancer types of the TCGA, to estimate how large is the set of drugs that are predicted to affect the different cancer types of the TCGA (see Supplementary 10)

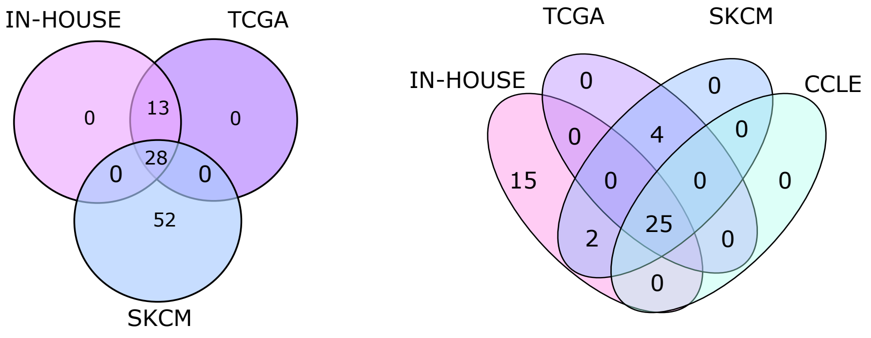

Figure S 8: **Shared essential drugs in the consensus (left) and sample-specific (right) models.** The drug overlap between the different conditions is shown in the Venn diagrams. Many drugs are specific to the SKCM and melanoma in the consensus and sample-specific models, respectively.

Among all the predicted drugs that have an inhibitory effect on the biomass production across the consensus TCGA models, 54 to 184 drugs are shared between CESC (Cervical Squamous Cell Carcinoma), SKCM (Skin Cutaneous Melanoma), BLCA (Urothelial Blad-

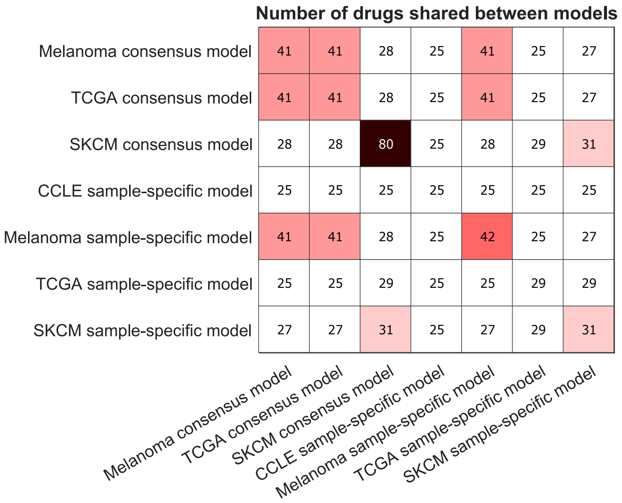

Figure S 9: **Shared drugs between the consensus and sample-specific cancer models.** The number of shared drugs is displayed for every cancer consensus and sample-specific model. The largest number of drugs was found for the SKCM consensus model whereas only 31 drugs were shared with the sample-specific models. The melanoma consensus and sample-specific models predicted the same drugs. The lowest number of drugs was predicted by the CCLE model, but they were also predicted by every other model.

der Carcinoma), LAML (Acute Myeloid Leukaemia), STAD (Stomach Adenocarcinoma), and UCS (Uterine Carcinosarcoma). Another cluster in which cancer types share 40 to 52 drugs can be observed that includes LGG (Low Grade Glioma), ACC (Adenoid cystic carcinoma), COAD (Colon adenocarcinoma), GBM (Glioblastoma multiforme), PRAD (Prostate adenocarcinoma), LIHC (Liver hepatocellular carcinoma), OV (Ovarian serous cystadenocarcinoma), THCA (Thyroid carcinoma), READ (Rectum adenocarcinoma), and KICH (Kidney Chromophobe). Because the same drugs have been predicted among these clusters, similar metabolic rewiring strategies that consequently lead to similar metabolic weaknesses can be suggested.

Some predicted drug predictions were already FDA approved for cancer

As an additional quality control, cancer data from drug databases were mined. 12 of the 28 candidate drugs have already been approved for antineoplastic therapy in at least one of the

	cancer DLBC	cancer STAD	cancer KIRP	cancer HNSC	cancer KIRC	cancer LUSC	cancer UCEC	cancer LUAD	cancer BRCA	cancer LGG	cancer ACC	cancer COAD	cancer GBM	cancer PRAD	cancer LIHC	cancer OV	cancer THCA	cancer READ	cancer KICH	cancer CESC	cancer SKCM	cancer BLCA	cancer LAML	cancer UCS
cancer DLBC	13	8	6	8	7	8	8	11	13	7	8	8	11	11	11	12	10	12	11	9	12	12	9	9
cancer STAD	8	59	9	10	10	11	11	9	14	10	10	11	10	10	10	10	10	11	10	59	59	59	37	59
cancer KIRP	6	9	26	25	26	26	26	26	26	25	25	26	25	25	25	25	26	26	26	26	26	26	26	26
cancer HNSC	8	10	25	28	26	27	27	26	28	27	28	27	27	27	27	28	25	27	26	28	27	27	28	28
cancer KIRC	7	10	26	26	42	26	26	27	30	27	26	26	25	25	26	26	28	26	29	28	27	27	30	28
cancer LUSC	8	11	26	27	26	29	29	27	28	27	27	29	27	27	27	27	26	28	26	29	28	29	28	28
cancer UCEC	8	11	26	27	26	29	29	27	28	27	27	29	27	27	27	27	26	28	26	29	28	29	28	28
cancer LUAD	11	9	26	26	27	27	27	32	31	25	26	27	29	29	29	30	30	30	31	28	30	31	27	27
cancer BRCA	13	14	26	28	30	28	28	31	45	27	28	28	31	31	31	32	38	32	39	33	35	35	33	32
cancer LGG	7	10	25	27	27	27	27	25	27	42	40	40	40	40	40	40	38	40	38	28	27	27	41	40
cancer ACC	8	10	25	28	26	27	27	26	28	40	41	40	40	40	40	41	38	40	39	29	27	27	41	41
cancer COAD	8	11	26	27	26	29	29	27	28	40	40	42	40	40	40	40	39	41	39	30	28	29	41	41
cancer GBM	11	10	25	27	25	27	27	29	31	40	40	40	44	44	44	44	42	44	42	28	31	31	40	40
cancer PRAD	11	10	25	27	25	27	27	29	31	40	40	40	44	44	44	44	42	44	42	28	31	31	40	40
cancer LIHC	11	10	25	27	26	27	27	29	31	40	40	40	44	44	46	44	42	44	42	28	31	31	40	40
cancer OV	12	10	25	28	26	27	27	30	32	40	41	40	44	44	44	45	42	44	43	29	31	31	41	41
cancer THCA	10	10	26	25	28	26	26	30	38	38	38	39	42	42	42	42	51	43	51	29	31	31	40	40
cancer READ	12	11	26	27	26	28	28	30	32	40	40	41	44	44	44	44	43	45	43	29	32	32	41	41
cancer KICH	11	10	26	26	29	26	26	31	39	38	39	39	42	42	42	43	51	43	52	30	31	31	41	41
cancer CESC	9	59	26	28	28	29	29	28	33	28	29	30	28	28	28	29	29	29	30	85	76	77	56	78
cancer SKCM	12	59	26	27	27	28	28	30	35	27	27	28	31	31	31	31	31	32	31	76	80	80	54	76
cancer BLCA	12	59	26	27	27	29	29	31	35	27	27	29	31	31	31	31	31	32	31	77	80	127	54	76
cancer LAML	9	37	26	28	30	28	28	27	33	41	41	41	40	40	40	41	40	41	41	56	54	54	148	68
cancer UCS	9	59	26	28	28	28	28	27	32	40	41	41	40	40	40	41	40	41	41	78	76	76	68	90

Figure S 10: **Shared predicted drugs across the different cancer tissues of the TCGA based on the consensus models.** The number of shared drugs between the different tissue-specific consensus models is shown. A cluster of cancer tissues (LAML, BLCA, UCS, SKCM, and CESC) with a high number of predicted drugs (54 to 148 drugs) that are also partially shared can be observed as well as another cluster (LGG, ACC, COAD, GBM, PRAD, LIHC, OV, THCA, READ and KICH) that share between 38 and 52 drugs can be observed, suggesting similar cancer liabilities in these clusters.

investigated databases (see Supplementary File 2). Further, only one database (SEER*RX) returned a significant enrichment for known antimetabolites (p-value: 0.0091), chemotherapeutic agents (p-value: 0.0300), and both (p-value: 0.0025), while no significant enrichment was found in other tested databases (see Methods). One possible explanation for this is the specificity of the predicted drugs that only target metabolic genes, whereas the databases include all the possible cancer drugs, which is further shown by the low number of found to target metabolic genes included in the Recon 2.04 reconstruction. Interestingly, no enrichment was found for melanoma-related anti-cancer agents in the SEER*Rx database, suggesting that we have predicted novel drugs for repurposing in melanoma, see Supplementary Table S3.

Table 3: **Drug Enrichments for the 28 candidate drugs.** Recon drugs is the number of drugs associated with any of the genes present in Recon2, Recon cancer drugs is the number of cancer drugs found in the database. The Drug list contains 28 potential drugs for repurposing in melanoma that showed inhibiting effects in the *in silico* analysis. Druglist cancer is the number of drugs associated with cancer in the given database. Using a hypergeometric test, we determined the p-value for each database.

Database	p-value	Recon drugs	Recon cancer drugs	Druglist	Druglist cancer
SEERRx all	0.0025	1175	220	28	11
SEERRx all alternate	0.0055	1175	274	28	12
SEERRx antimetabolites	0.0091	1175	19	28	2
SEERRx chemotherapy	0.0124	1175	85	28	5
SEERRx chemotherapy alternate	0.0300	1175	103	28	5
CancerResearch UK	0.0501	1175	87	28	4
navigationgcare	0.0794	1175	99	28	4
Chemocare	0.1199	1175	112	28	4
SEERRx melanoma	0.1350	1175	6	28	0
SEERRx skin	0.1350	1175	6	28	0
SEERRx skin alternate	0.1350	1175	6	28	0
SEERRx melanoma alternate	0.1760	1175	8	28	0
CancerGov	0.2433	1175	72	28	2
centerwatch	0.4491	1175	63	28	1

Predicted drugs and their targets show higher viability reduction and dependency, respectively, than anti-melanoma drugs and their targets, in both resistant and metastatic melanoma cell lines

The viability reduction were retrieved for 25 predicted, 9 anti-melanoma and 29 NO-based drugs from the primary PRISM database. Aminomethyltransferase enzyme (https://clue.io/repurposing-app?q=Name:aminomethyltransferase) was mislabeled as a compound in the Drug Repurposing Hub: thus, it was discarded from the NO-based drugs. Three predicted drugs (cladribine, fluvastatin, and gemcitabine) ranked higher than anti-melanoma drugs in both metastatic (Supplementary Figure S11) and resistant cell lines (Supplementary Figures S12 and S13). Among the 29 NO-based drugs, diphenyleneiodonium is the only drug with a median viability reduction higher than 50% in either metastatic or resistant cell lines. The high ranking of the non-anti-cancer drug, fluvastatin, among the predicted anti-cancers (gemcitabine and cladribine) in both metastatic and resistant cell lines, underlines fluvastatin as a potential anti-melanoma drug.

In both metastatic and resistant cell lines, predicted drug main targets have stronger dependency than anti-melanoma targets, with RRM1 and RRM2 scoring the highest, see Supplementary Figures S14 and S15. Main drug targets (anti-melanoma and predicted drugs) were selected from the drug targets using using the Drug Repurposing Hub (Corsello et al., 2017) for its high manual curation. Unlike the primary PRISM database, DepMap offers eight drug-induced resistance cell lines that are corresponding to four wildtype cell lines. ASS1 was the highest NO-related gene with a 30% dependency probability in metastatic cell lines. As DepMap shows the effect of single gene knock-out in vitro in cell lines, thus potential combination effect of NO-related genes with other targets is beyond this database and would need further testing.

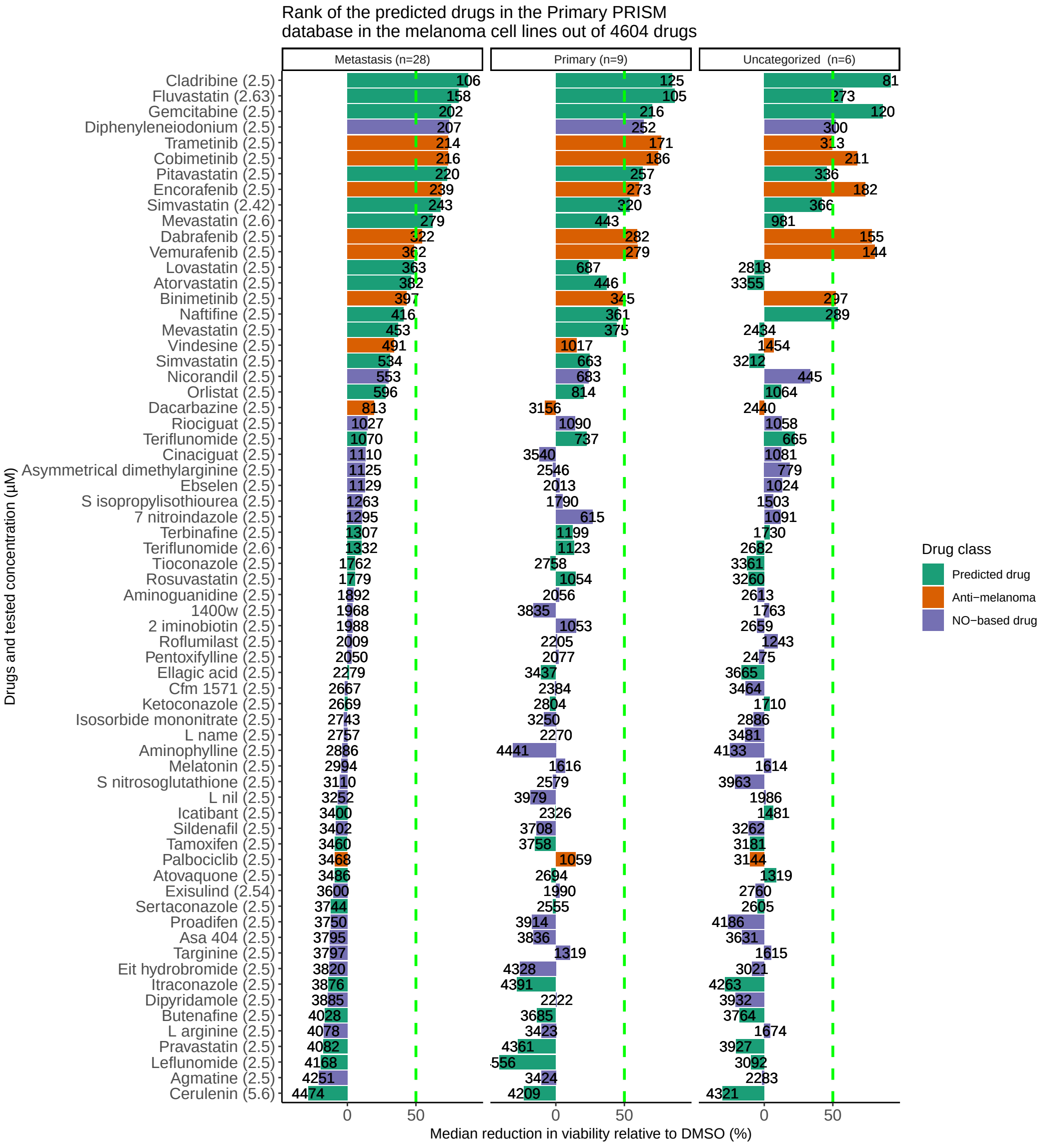

Figure S 11: **Cladribine, fluvastatin and gemcitabine and the NO-based drug diphenyleneiodonium rank better than anti-melanoma drugs in viability reduction assays.** The viability reduction relative to DMSO for our 28 predicted, anti-melanoma and NO-based drugs were gathered from the primary PRISM database for metastatic, primary, and uncategorized cell lines. The drugs were ranked by their median viability reduction in metastatic cell lines. X-axis represents the median viability reduction relative to DMSO (%) for metastatic (left), primary (middle) and uncategorized (right) cell lines. The rank of each drug out of the 4606 tested drugs in the primary PRISM database was printed beside each bar.

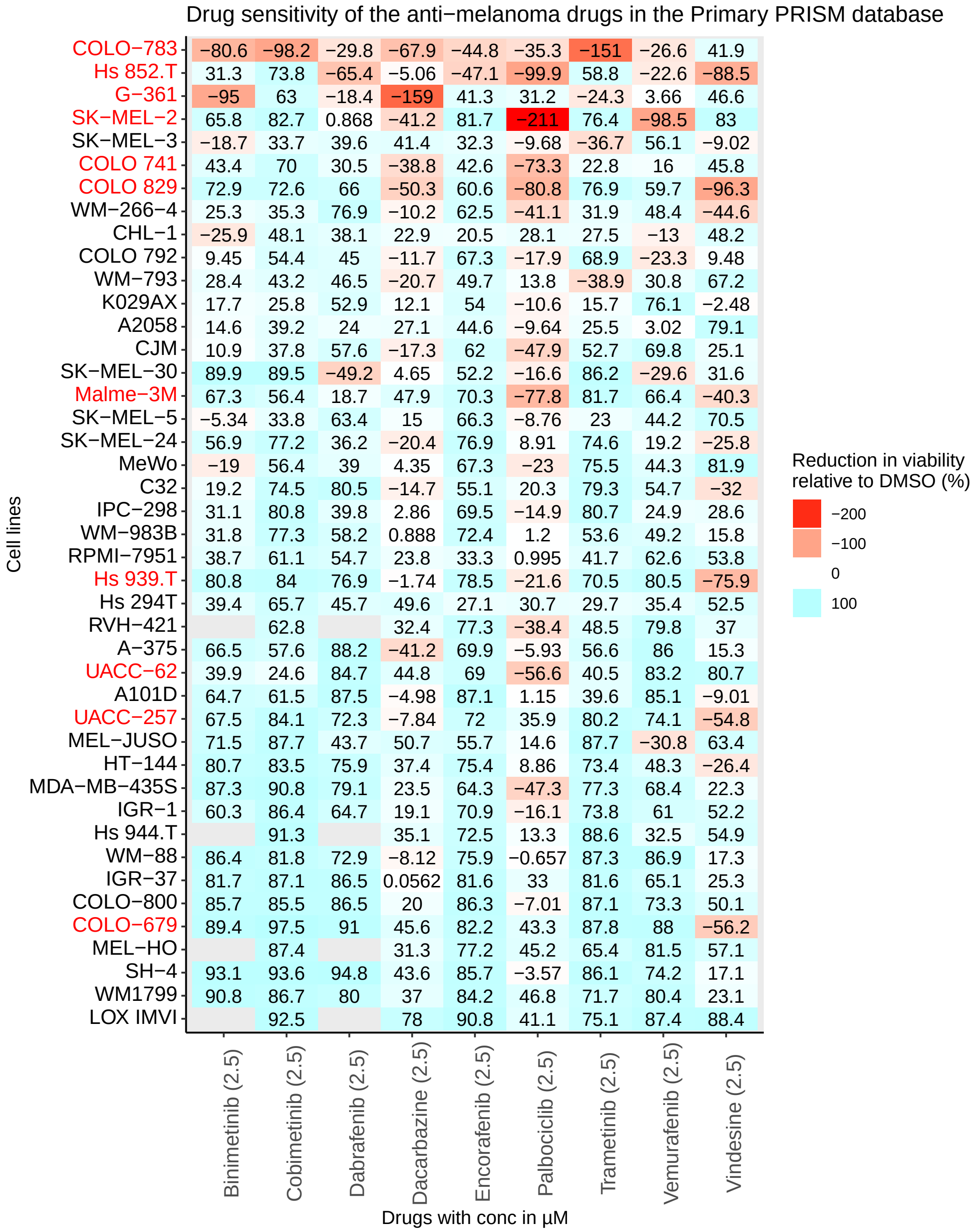

Figure S 12: **Over a quarter of the melanoma cell lines displayed resistance to anti-melanoma drugs in the primary PRISM database.** Effect of drugs ranges from viability reduction (in blue) to increase of proliferation (up to more than 2-fold increase, in red). Cell lines with at least 50% increased proliferation (-50% viability reduction) to any anti-melanoma drug, were considered resistant cell lines (names highlighted in red) for visualisation in Figure S13. The x-axis represents the anti-melanoma drugs and the y-axis represents the melanoma cell lines. Missing drug-cell line experiments are marked in grey tiles.

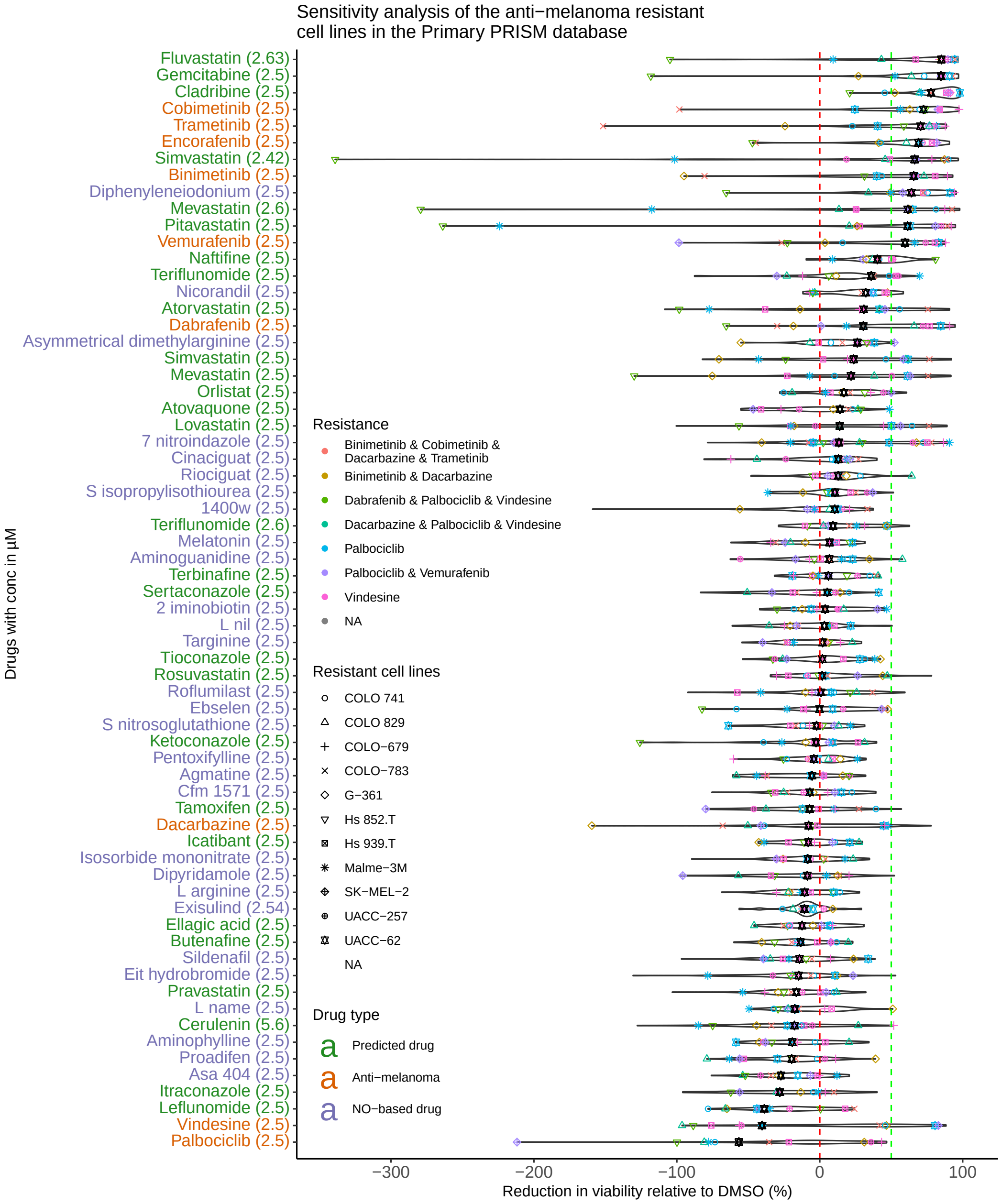

Figure S 13: **Fluvastatin, gemcitabine and cladribine outperformed anti-melanoma and NO-drugs on anti-melanoma drug resistant cell lines.** Cell lines annotated as resistant to anti-melanoma drugs in the primary PRISM database (with viability reduction >-50%, names highlighted in red), were used to rank all drugs by their median viability reduction (black dot). The violin plot represents the viability reduction for different drugs across all melanoma cell lines of the primary PRISM database. Predicted, anti-melanoma, and NO-based drugs' names on the y-axis have green, orange and violet colours, respectively. The resistant melanoma cell lines highlighted by marker. The marker shape indicates the cell line and the marker colour indicates the drug the cell line is resistant to.

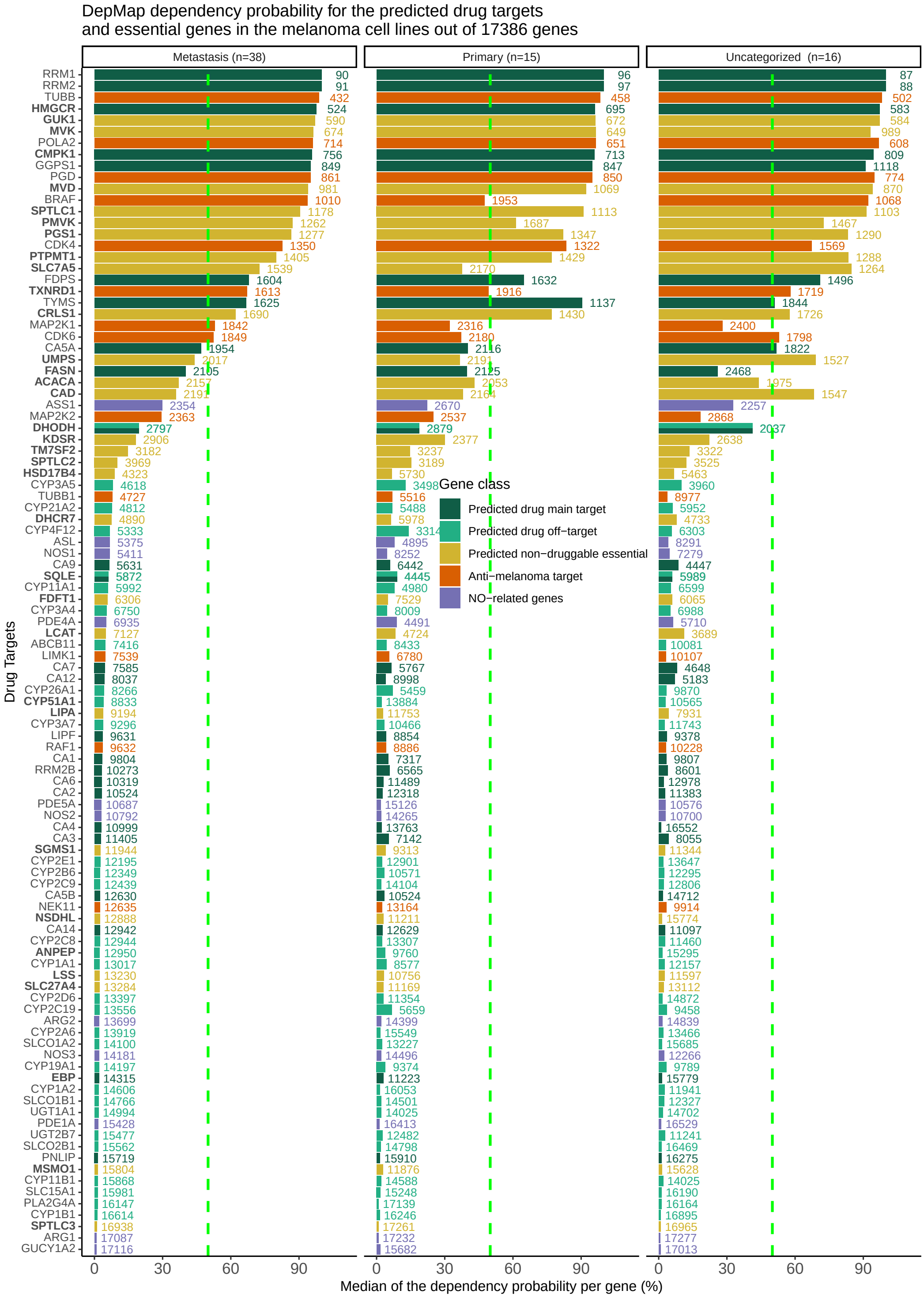

Figure S 14: **The knockout of the predicted drug targets and essential genes induced a stronger viability reduction than the melanoma targets and NO-related genes.** Predicted drug targets and essential genes were compared to both the anti-melanoma targets and NO-related genes based on gene dependency probability scores (probability to induce cell death or stop cell growth upon knock-out) from DepMap in metastatic melanoma cell lines. X-axis represents the median dependency probability across three types of melanoma cell lines (metastasis, primary, and uncategorized). Essential genes are highlighted in bold and the non-druggable essential genes (according to DrugBank v5.1.3) have yellow bars. Genes are ranked on the y-axis by the median dependency probability in the metastatic cell lines, with the gene rank displayed on the right of each bar.

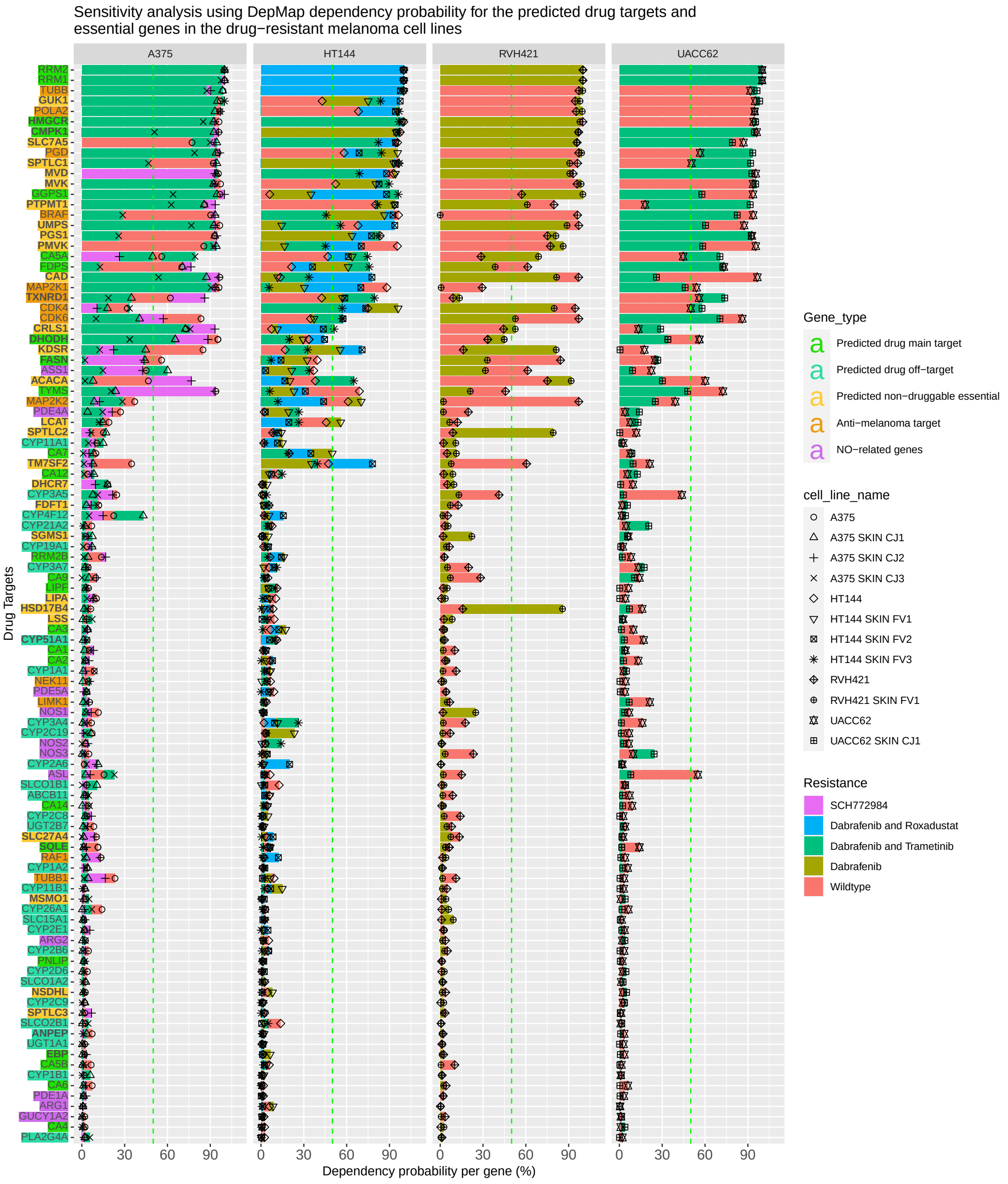

Figure S 15: **The knockout of predicted essential genes induce a higher viability reduction in resistant cell lines.** The dependency probability of the predicted drug targets and essential genes were compared to anti-melanoma targets and NO-related genes using the drug-induced resistance (violet, brown, green and blue bars) and wildtype (red bars) cell lines from the DepMap database. Genes (predicted drug targets, essential genes, anti-melanoma targets and NO-related genes) were sorted by the median dependency probability across the eight resistant cell lines.

Gemcitabine and cladribine show low IC_{50} under 0.4 μM as seven of the ten anti-melanoma drugs in melanoma cell lines from high-throughput drug viability databases.

The merged $databaseincluded1804drugsand1490celllines.ThemergedIC_{50}$ database retrieved 10 anti-melanoma, 13 predicted, and 4 NO-based drugs with variant numbers of melanoma cell lines per drug ranging from 1 to 63. Ranking with the median IC_{50} showed seven of the nine anti-melanoma drugs were below 0.4 μM, see Supplementary Figure S16. Gemcitabine and cladribine of the predicted drugs and only diphenyleneiodonium of the NO-based drugs were below the 0.4 μM threshold. Moreover, two predicted statins (pitavastatin and fluvastatin) were below 1 μM median IC_{50}. Some anti-melanoma drugs, especially BRAF-inhibitors (encorafenib dabrafenib) showed higher resistance in BRAF-wildtype and NRAS-mutant. Meanwhile, the predicted drugs, especially cladribine, the median IC_{50} remained unaffected regardless of the BRAF or NRAS mutation, see Supplementary Figure S17.

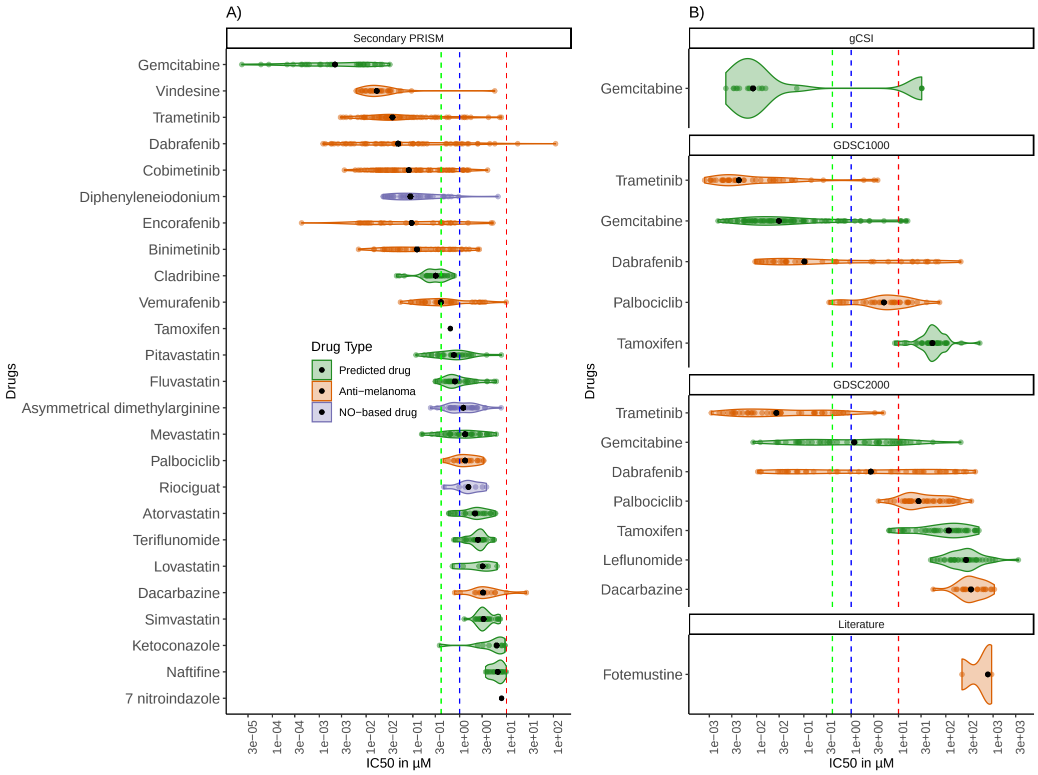

Figure S 16: **Gemcitabine, cladribine and diphenyleneiodonium had comparable IC_{50} value data to known anti-melanoma drugs in melanoma cell lines in the secondary PRISM database.** Four databases (Secondary PRISM, GDSC1000, GDSC2000 and gSCI) of cell viability in cancer cell lines with IC_{50} values were merged for the tested drugs across melanoma cell lines. Black dots represent the median IC_{50}. Green, blue and red dashed lines correspond to 0.4, 1 and 10 μM, respectively. Seven of the ten anti-melanoma drugs have median IC_{50} below 0.4 μM (green line), whereas gemcitabine and cladribine have their median IC_{50} below this threshold of 0.4 μM.

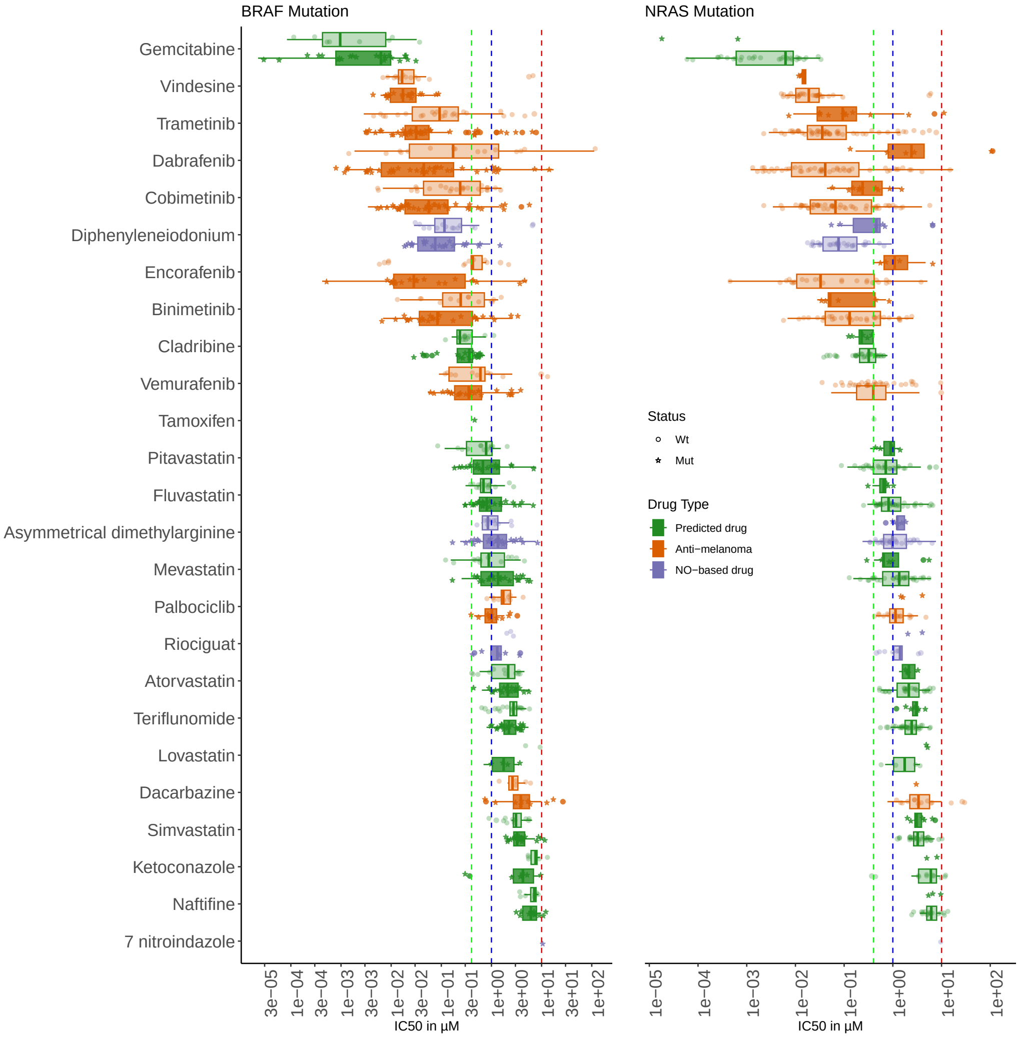

Figure S 17: **The IC_{50} values of cladribine and other predicted drugs unlike anti-melanoma drugs are independent of the BRAF or NRAS mutation status.** IC_{50} (Supplementary Figure S16 .A) were classified based on the mutation status in BRAF and NRAS genes. Transparent and opaque box plots correspond to wildtype and mutants, respectively.

1 *In vitro* validation

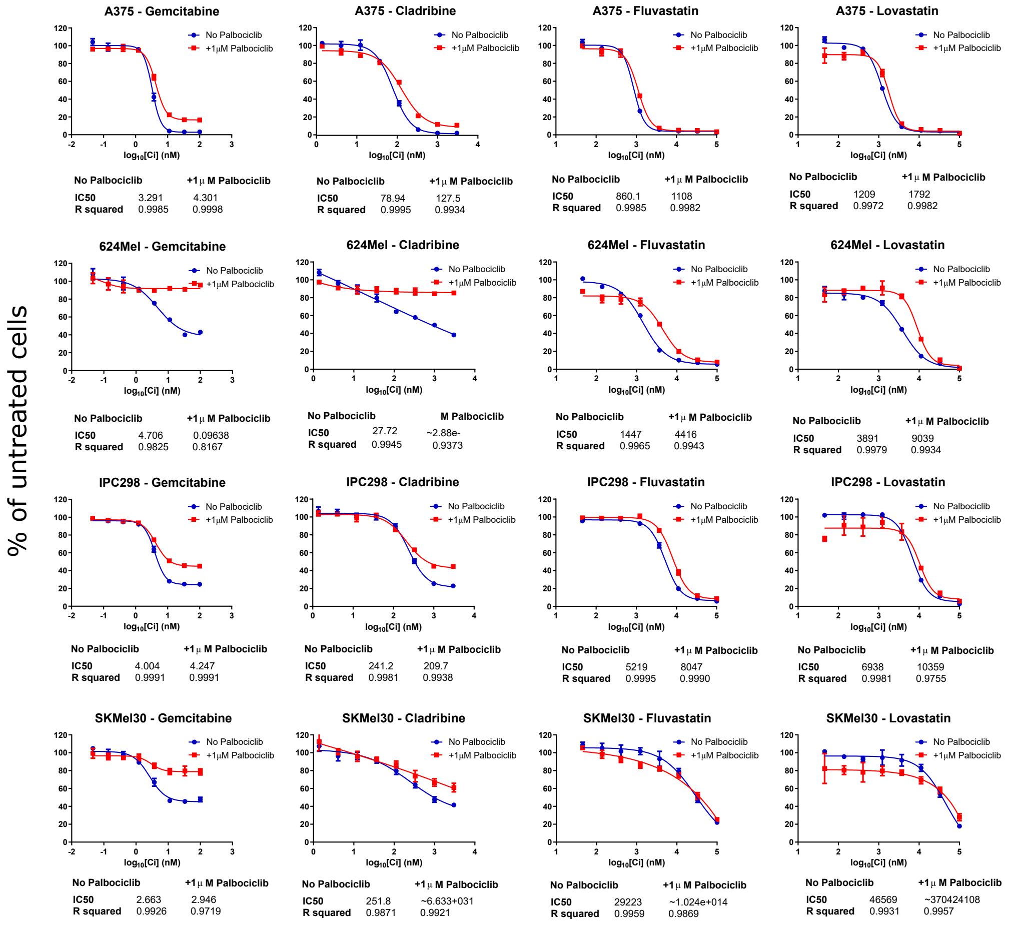

Figure S 18: IC_{50} **assays for CDK4/6i with four selected drugs.** Mostly antagonistic effects can be observed.

Table 4: **Mean IC_{50} values per drug.** For each drug and cell line, the mean IC_{50} value was determined. For some drugs, no IC_{50} was obtained because no effect was found on the cells.

	A375N		624Mel	
	Mean IC_{50} (nM)	StDev (nM)	Mean (nM)	StDev (nM)
Butenafine	-	-	-	-
Cerulenin	13713.8	592.3	14070.0	300.2
Ellagic acid	-	-	-	-
Fluvastatin	1034.5	64.3	2096.3	93.0
Icatibant	-	-	-	-
Terbinafine	-	-	-	-
Atovaquone	-	-	-	-
Cladribine	125.7	18.2	1433.2	1124.6
Gemcitabine	4.8	0.3	34.1	24.9
Lovastatin	1639.7	56.1	5263.3	153.7
Tamoxifen	-	-	5675.5	402.9
Tioconazole	-	-	-	-

	IPC298		SKMel30	
	Mean (nM)	StDev (nM)	Mean (nM)	StDev (nM)
Butenafine	-	-	-	-
Cerulenin	15498.8	962.9	17224.3	1097.6
Ellagic acid	-	-	-	-
Fluvastatin	5976.0	244.5	22191.8	2579.3
Icatibant	-	-	-	-
Terbinafine	-	-	-	-
Atovaquone	-	-	-	-
Cladribine	530.2	137.7	1009.7	624.6
Gemcitabine	4.7	0.5	22.8	15.4
Lovastatin	7302.3	442.8	-	-
Tamoxifen	11483.3	675.8	10747.3	510.6
Tioconazole	-	-	-	-

Table 5: **Synergy scores for BRAFi and four predicted drugs for repurposing**. Synergy experiments were performed for the different drug combinations on the BRAF-mutated cell lines.

**A375N**	Gemcitabine		Cladribine		Fluvastatin		Lovastatin		Dilution Factor
Vemurafenib	Add	4.1	Add	7.11	Add	6.18	Add	5.48	1:2
	Add	2.96	Add	3.38	Syn	13.08	Add	6.48	1:1,5
Dabrafenib	Add	6.32	Add	4.81	Add	7.25	Add	6.07	1:2
	Add	2.51	Add	1.53	Add	7.32	Add	6.84	1:1,5
Encorafenib	Add	4.03	Add	8.33	Syn	11.25	Add	5.12	1:2
	Add	1.8	Add	3.12	Add	6.75	Add	4.81	1:1,5

**624Mel**	Gemcitabine		Cladribine		Fluvastatin		Lovastatin		Dilution Factor
Vemurafenib	Add	5.28	Add	6.54	Add	6.59	Add	3.75	1:2
	Syn	13.98	Syn	11.72	Syn	15.43	Syn	10.46	1:1,5
Dabrafenib	Add	7.14	Add	8.22	Add	5.12	Add	4.87	1:2
	Add	9.75	Syn	13.93	Syn	11.08	Syn	11.83	1:1,5
Encorafenib	Add	9.25	Add	9.39	Add	8.64	Add	6.45	1:2
	Syn	11.43	Syn	12.86	Syn	13.4	Add	9.33	1:1,5

Table 6: **Synergy scores for MEKi and four predicted drugs for repurposing**. Synergy experiments were performed for the different drug combinations on four different cell lines. Due to a low IC_{50} value in the previous experiment, lovastatin was not used for the SKMel30 cell line.

**A375N**	Gemcitabine		Cladribine		Fluvastatin		Lovastatin		Dilution Factor
Binimetinib	Add	7.29	Add	6.29	Add	6.64	Add	4.89	1:2
	Add	4.38	Add	2.52	Add	9.42	Syn	10.9	1:1,5

**624Mel**	Gemcitabine		Cladribine		Fluvastatin		Lovastatin		Dilution Factor
Binimetinib	Add	8.34	Syn	11.59	Add	7.53	Add	5.24	1:2
	Add	5.07	Syn	13.06	Add	5.51	Add	4.45	1:1,5

**IPC298**	Gemcitabine		Cladribine		Fluvastatin		Lovastatin		Dilution Factor
Binimetinib	Add	6.64	Add	7.46	Add	4.9	Add	4.4	1:2
	Syn	14.77	Syn	11.74	Add	1.98	Add	3.78	1:1,5

**SKMel30**	Gemcitabine		Cladribine		Fluvastatin				Dilution Factor
Binimetinib	Syn	10.59	Add	7.03	Add	4.79			1:2
	Syn	12.08	Add	9.12	Syn	10.21			1:1,5

PrestoBlue Assay: Proliferation Assay

Different drugs with Binimetinib (MEKi)

624Mel

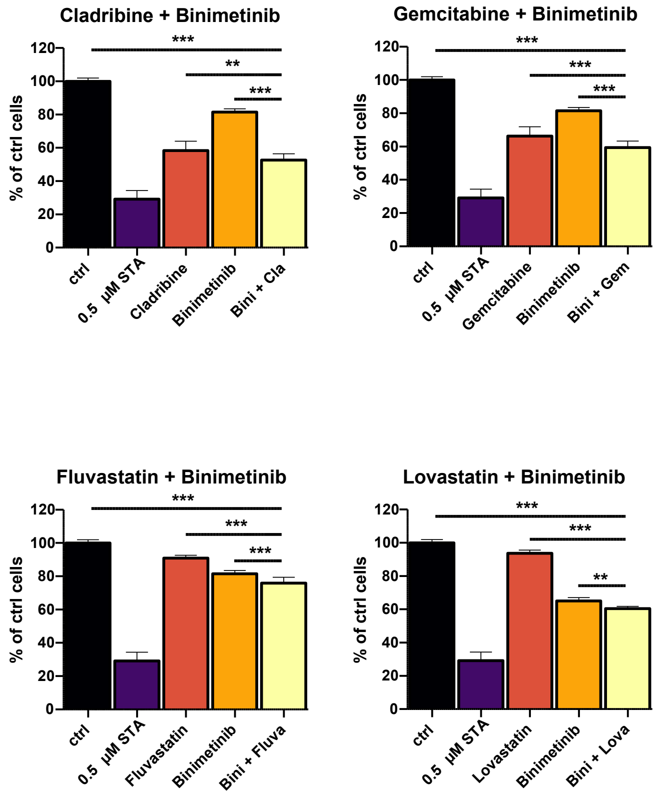

SKMel30

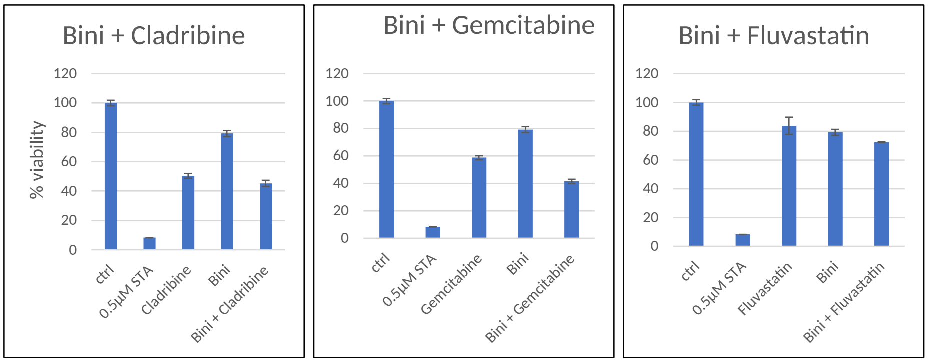

1 representative exp.

Figure S 19: **Proliferation assay of binimetinib (MEKi) with four predicted drugs in 624Mel (BRAF mutant) and SKMel30 (NRAS mutant) cell lines.**

Propidium Iodide Staining: Dead Cell Count

Different drugs with Binimetinib (MEKi)

624Mel

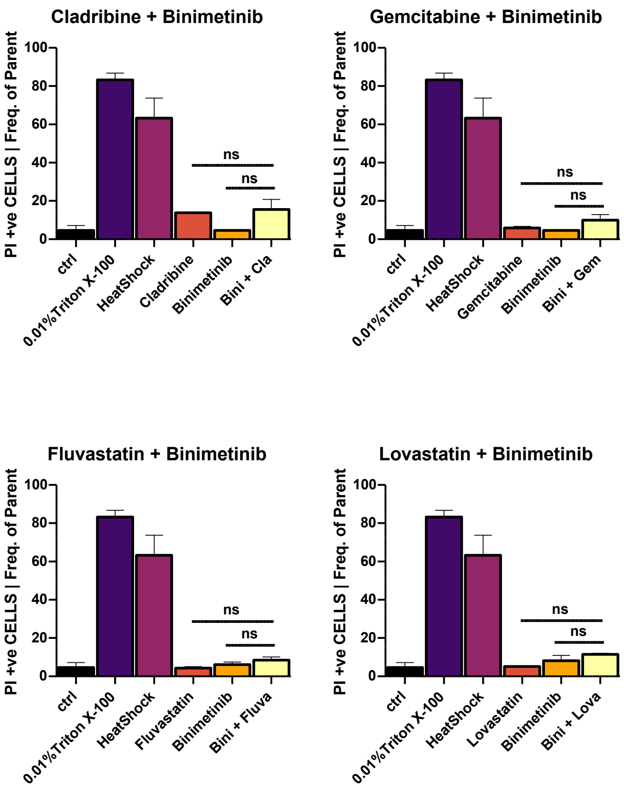

SKMel30

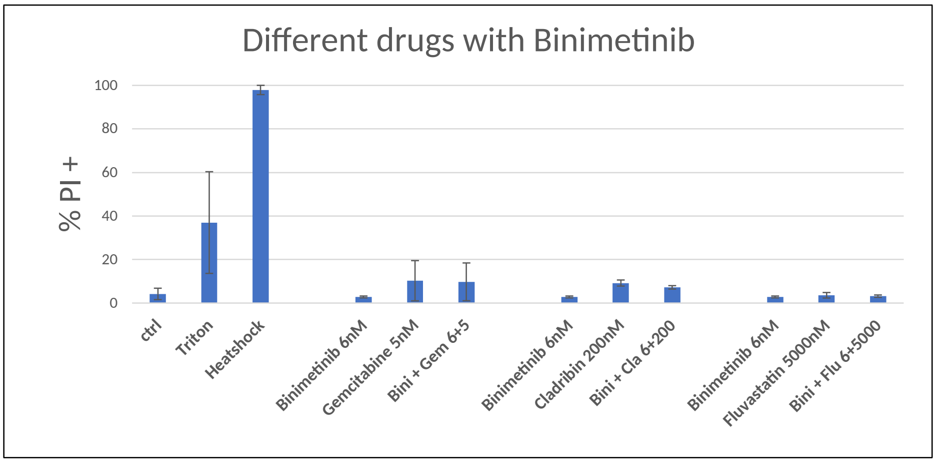

Figure S 20: **PI dead cell staining of binimetinib (MEKi) with four predicted drugs in 624Mel (BRAF mutant) and SKMel30 (NRAS mutant) cell lines.**

Ac-DEVD Caspase 3/7 Assay: Apoptosis Assay

Different drugs with Binimetinib (MEKi)

624Mel

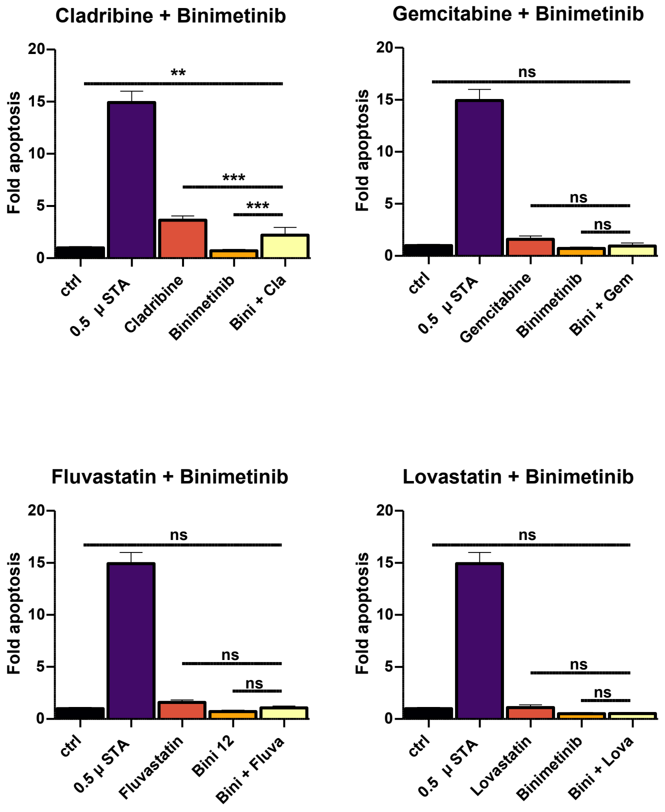

SKMel30

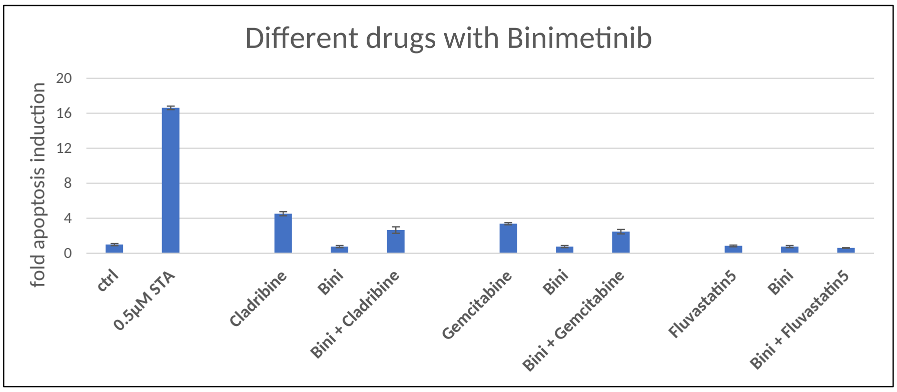

1 representative exp.

Figure S 21: **Caspase 3/7 Ac-DEVD-AFC apoptosis assay of binimetinib (MEKi) with four predicted drugs in 624Mel (BRAF mutant) and SKMel30 (NRAS mutant) cell lines.**

PrestoBlue Assay: Proliferation Assay

Different drugs with PLX4032 (BRAFi)

624Mel

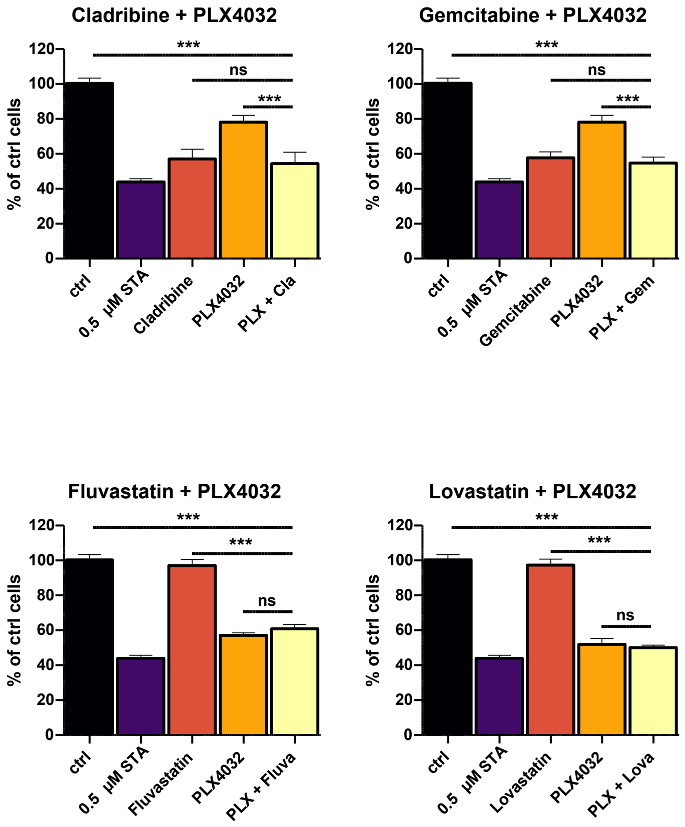

SKMel30

No data for SKMel30 cells: they do not react to BRAF inhibitors, because they have an NRAS but not a BRAF mutation

Figure S 22: **Proliferation assay of vemurafenib (BRAFi) with four predicted drugs in 624Mel (BRAF mutant) and SKMel30 (NRAS mutant) cell lines.**

Propidium Iodide Staining: Dead Cell Count

Different drugs with PLX4032 (BRAFi)

624Mel

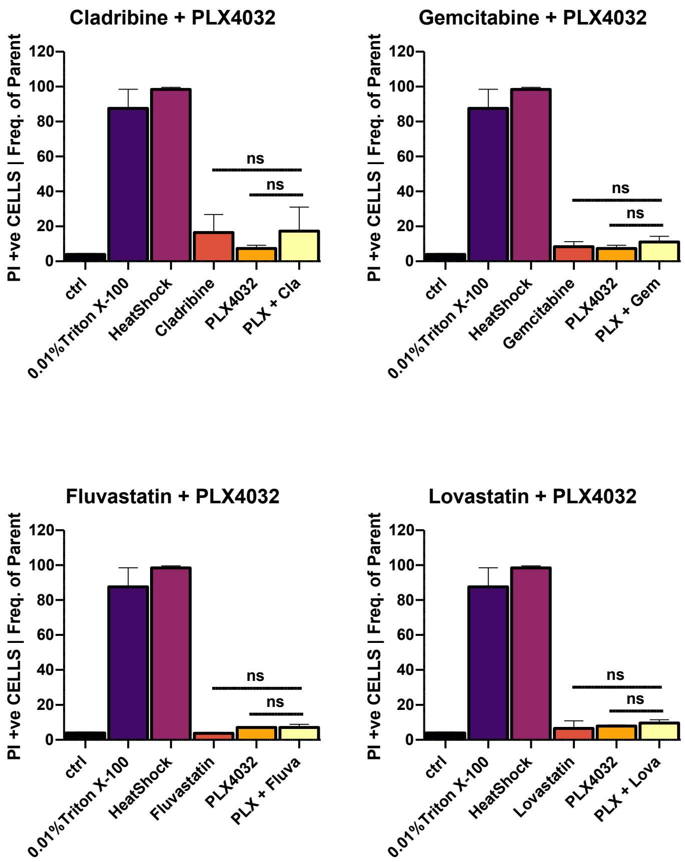

SKMel30

No data for SKMel30 cells: they do not react to BRAF inhibitors, because they have an NRAS but not a BRAF mutation

Figure S 23: **PI dead cell staining of vemurafenib (BRAFi) with four predicted drugs in 624Mel (BRAF mutant) and SKMel30 (NRAS mutant) cell lines.**

Ac-DEVD Caspase 3/7 Assay: Apoptosis Assay

Different drugs with PLX4032 (BRAFi)

624Mel

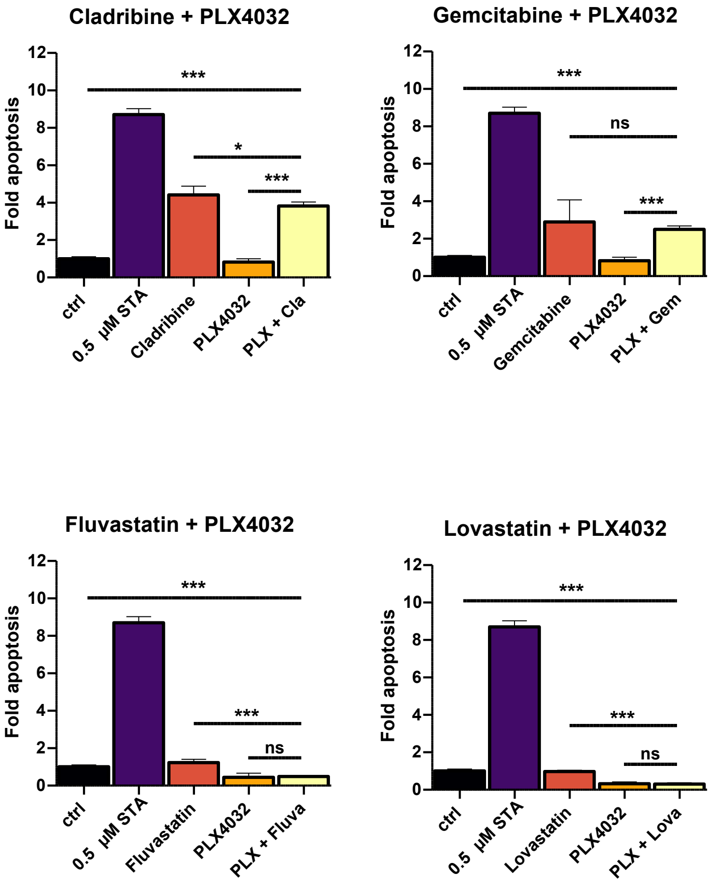

SKMel30

No data for SKMel30 cells: they do not react to BRAF inhibitors, because they have an NRAS but not a BRAF mutation

Figure S 24: **Gemcitabine and cladribine induce apoptosis. However the combination with PLX4032 (BRAFi) on the 624Mel cell line did not show any additive effect.**

PrestoBlue Assay: Proliferation Assay

Different drugs with Palbociclib (CDK4/6i)

624Mel

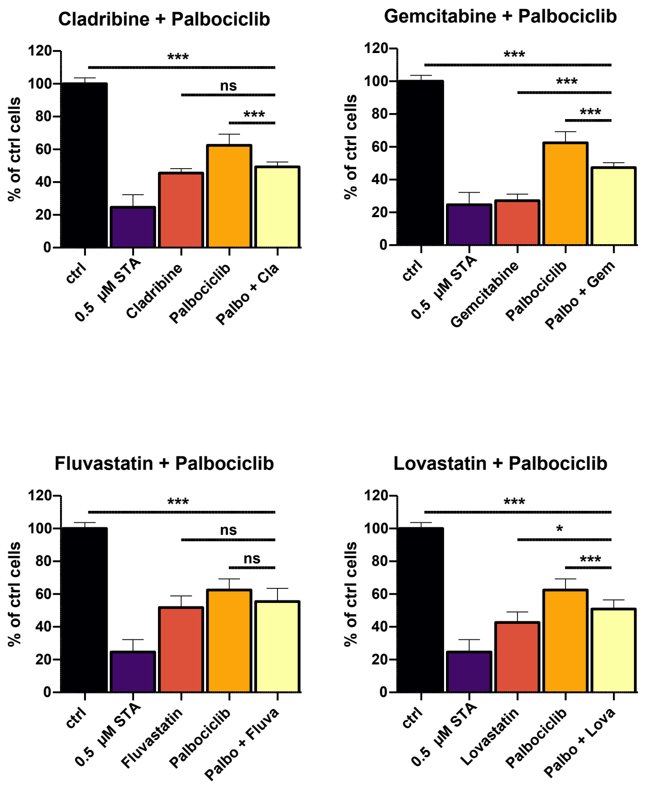

SKMel30

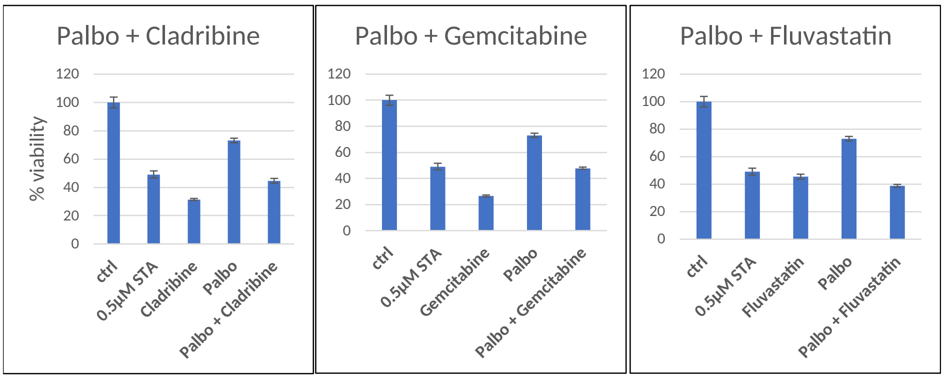

1 representative exp.

Figure S 25: **Proliferation assay of palbociclib (CDK4/6i) with four predicted drugs in 624Mel (BRAF mutant) and SKMel30 (NRAS mutant) cell lines.**

Propidium Iodide Staining: Dead Cell Count

Different drugs with Palbociclib (CDK4/6i)

624Mel

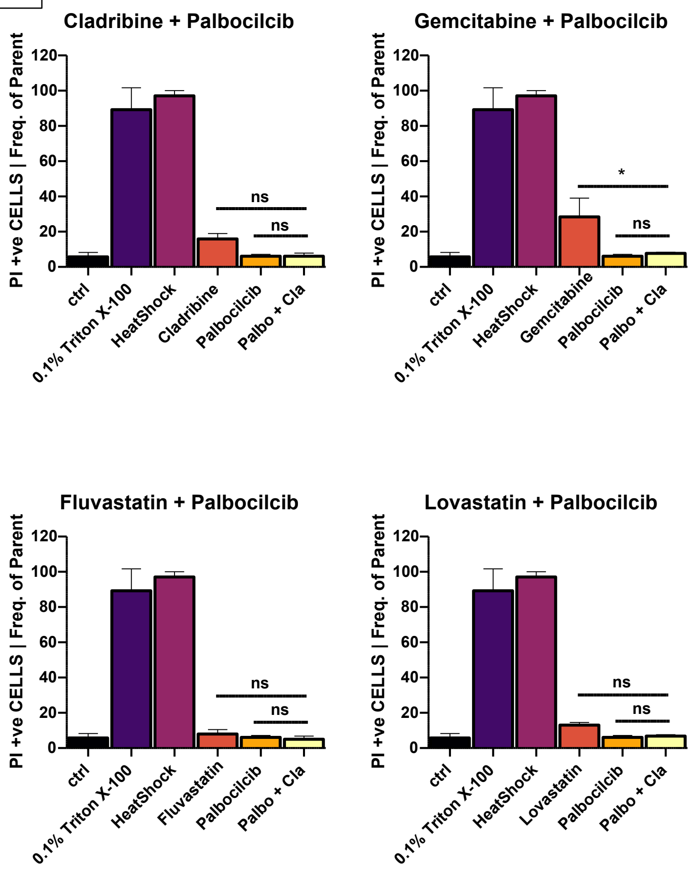

SKMel30

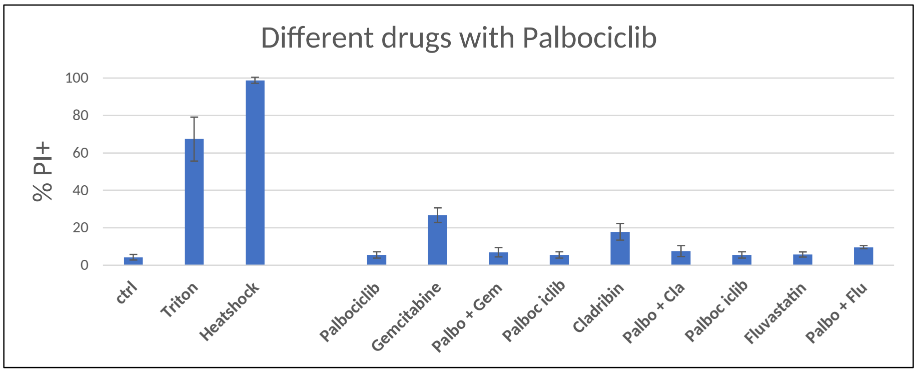

Figure S 26: **PI dead cell staining of palbociclib (CDK4/6i) with four predicted drugs in 624Mel (BRAF mutant) and SKMel30 (NRAS mutant) cell lines.**

Ac-DEVD Caspase 3/7 Assay: Apoptosis Assay

Different drugs with Palbociclib (CDK4/6i)

624Mel

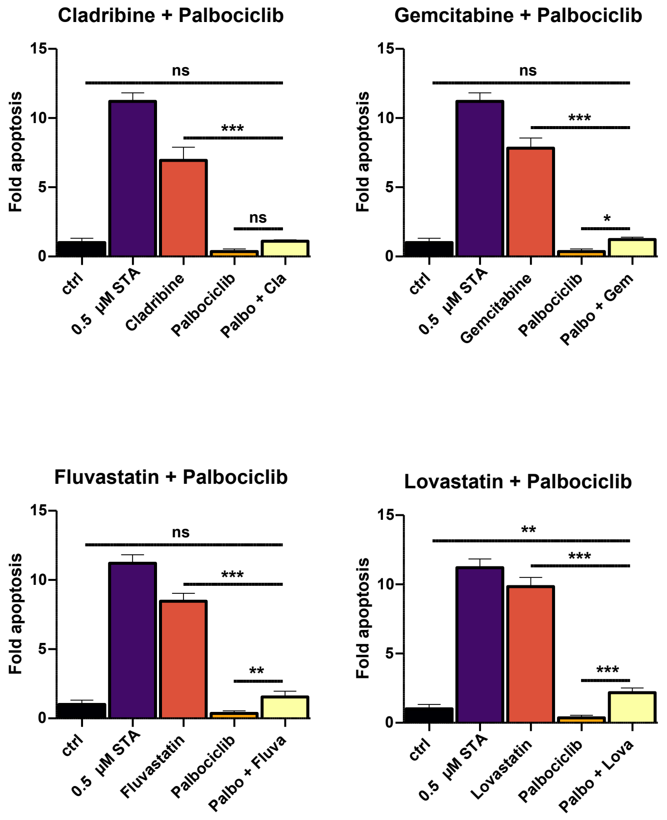

SKMel30

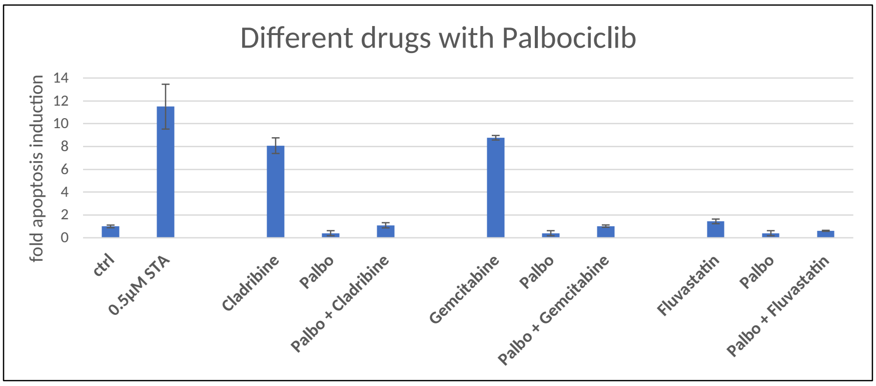

1 representative exp.

Figure S 27: **Caspase 3/7 Ac-DEVD-AFC apoptosis assay of palbociclib (CDK4/6i) with four predicted drugs in 624Mel (BRAF mutant) and SKMel30 (NRAS mutant) cell lines.**

References

Corsello, S. M., Bittker, J. A., Liu, Z., Gould, J., McCarren, P., Hirschman, J. E., Johnston, S. E., Vrcic, A., Wong, B., Khan, M., Asiedu, J., Narayan, R., Mader, C. C., Subramanian, A., and Golub, T. R. (2017). The Drug Repurposing Hub: a next-generation drug library and information resource. *Nature Medicine*, 23(4):405–408.

Corsello, S. M., Nagari, R. T., Spangler, R. D., Rossen, J., Kocak, M., Bryan, J. G., Humeidi, R., Peck, D., Wu, X., Tang, A. A., Wang, V. M., Bender, S. A., Lemire, E., Narayan, R., Montgomery, P., Ben-David, U., Garvie, C. W., Chen, Y., Rees, M. G., Lyons, N. J., McFarland, J. M., Wong, B. T., Wang, L., Dumont, N., O'Hearn, P. J., Stefan, E., Doench, J. G., Harrington, C. N., Greulich, H., Meyerson, M., Vazquez, F., Subramanian, A., Roth, J. A., Bittker, J. A., Boehm, J. S., Mader, C. C., Tsherniak, A., and Golub, T. R. (2020). Discovering the anti-cancer potential of non-oncology drugs by systematic viability profiling. *Nature cancer*, 1:235–248.

Dobin, A., Davis, C. A., Schlesinger, F., Drenkow, J., Zaleski, C., Jha, S., Batut, P., Chaisson, M., and Gingeras, T. R. (2013). STAR: ultrafast universal RNA-seq aligner. *Bioinformatics*, 29(1):15–21.

Haverty, P. M., Lin, E., Tan, J., Yu, Y., Lam, B., Lianoglou, S., Neve, R. M., Martin, S., Settleman, J., Yauch, R. L., and Bourgon, R. (2016). Reproducible pharmacogenomic profiling of cancer cell line panels. *Nature*, 533(7603):333–337.

Janakiram, N. B. and Rao, C. V. (2012). inos-selective inhibitors for cancer prevention: promise and progress. *Future medicinal chemistry*, 4(17):2193–2204.

Kim, D., Langmead, B., and Salzberg, S. L. (2015). HISAT: a fast spliced aligner with low memory requirements. *Nature methods*, 12(4):357–360.

Li, B. and Dewey, C. N. (2011). RSEM: accurate transcript quantification from RNA-Seq data with or without a reference genome. *BMC bioinformatics*, 12(1):1–16.

Liao, Y., Smyth, G. K., and Shi, W. (2019). The R package Rsubread is easier, faster, cheaper and better for alignment and quantification of RNA sequencing reads. *Nucleic acids research*, 47(8):e47—-e47.

Louveau, B., Resche-Rigon, M., Lesimple, T., Da Meda, L., Pracht, M., Baroudjian, B., Delyon, J., Amini-Adle, M., Dutriaux, C., Reger de Moura, C., et al. (2021). Phase I–II Open-Label Multicenter Study of Palbociclib+ Vemurafenib in BRAFV600MUT Metastatic Melanoma Patients: Uncovering CHEK2 as a Major Response MechanismPalbociclib+ Vemurafenib in Metastatic Melanoma. *Clinical Cancer Research*, 27(14):3876–3883.

Love, M. I., Huber, W., and Anders, S. (2014). Moderated estimation of fold change and dispersion for RNA-seq data with DESeq2. *Genome biology*, 15(12):1–21.

Margue, C., Philippidou, D., Kozar, I., Cesi, G., Felten, P., Kulms, D., Letellier, E., Haan, C., and Kreis, S. (2019). Kinase inhibitor library screening identifies synergistic drug combinations effective in sensitive and resistant melanoma cells. *Journal of Experimental & Clinical Cancer Research*, 38(1):1–17.

Mintz, J., Vedenko, A., Rosete, O., Shah, K., Goldstein, G., Hare, J. M., Ramasamy, R., and Arora, H. (2021). Current Advances of Nitric Oxide in Cancer and Anticancer Therapeutics. *Vaccines*, 9:1–39.

Pacheco, M. P., Bintener, T., Ternes, D., Kulms, D., Haan, S., Letellier, E., and Sauter, T. (2019). Identifying and targeting cancer-specific metabolism with network-based drug target prediction. *EBioMedicine*, 43:98–106.

Pacini, C., Dempster, J. M., Boyle, I., Gonçalves, E., Najgebauer, H., Karakoc, E., van der Meer, D., Barthorpe, A., Lightfoot, H., Jaaks, P., et al. (2021). Integrated cross-study datasets of genetic dependencies in cancer. *Nature communications*, 12(1):1–14.

Schubert, M., Ermini, L., Sarkissian, C. D., Jónsson, H., Ginolhac, A., Schaefer, R., Martin, M. D., Fernández, R., Kircher, M., McCue, M., et al. (2014). Characterization of ancient and modern genomes by SNP detection and phylogenomic and metagenomic analysis using PALEOMIX. *Nature protocols*, 9(5):1056–1082.

Schubert, M., Lindgreen, S., and Orlando, L. (2016). AdapterRemoval v2: rapid adapter trimming, identification, and read merging. *BMC research notes*, 9(1):1–7.

Stasch, J. P., Pacher, P., and Evgenov, O. V. (2011). Soluble Guanylate Cyclase as an Emerging Therapeutic Target in Cardiopulmonary Disease. *Circulation*, 123:2263.

Yang, W., Soares, J., Greninger, P., Edelman, E. J., Lightfoot, H., Forbes, S., Bindal, N., Beare, D., Smith, J. A., Thompson, I. R., Ramaswamy, S., Futreal, P. A., Haber, D. A., Stratton, M. R., Benes, C., McDermott, U., and Garnett, M. J. (2013). Genomics of Drug Sensitivity in Cancer (GDSC): a resource for therapeutic biomarker discovery in cancer cells. *Nucleic Acids Research*, 41(D1):D955—-D961.
