# Supplementary material for: Metabolic modelling-based in silico drug target prediction identifies six novel repurposable drugs for melanoma
Source: Cell Death Dis. 2023 Jul 26;14(7):468. doi: 10.1038/s41419-023-05955-1 (PMC10372000; doi:10.1038/s41419-023-05955-1)
Supplement: Supplementary file 4 — confirmCoauthorAliKishk [file 41419_2023_5955_MOESM4_ESM.pdf]

Thomas SAUTER

From: Demetra PHILIPPIDOU
Sent: Friday, June 30, 2023 11:08 AM
To: Lasse SINKKONEN; Thomas SAUTER
Cc: Stephanie KREIS; tamara_bintener@hotmail.com; Christiane WURTH - MARGUE; dmgreta@hotmail.it; luca@cancer.dk; Maria Isabel MOSCARDO GARCIA; Rashi HALDER; Daniela De Zio (dzio@cancer.dk); Kulms, Dagmar (Dagmar.Kulms@uniklinikum-dresden.de); Maria PACHECO
Subject: RE: Cell Death & Disease paper: co-author Ali Kishk

I confirm.

Demetra PHILIPPIDOU

Research Support Engineer

UNIVERSITY OF LUXEMBOURG
Faculty of Science, Technology and Medicine
Department of Life Sciences and Medicine
Signal Transduction Laboratory
6, avenue du Swing
L-4367 Belvaux

Tel.: (+352) 46 66 44 **6167**
E-mail: demetra.philippidou@uni.lu

From: Lasse SINKKONEN <lasse.sinkkonen@uni.lu>
Sent: 29 June 2023 17:17
To: Thomas SAUTER <Thomas.Sauter@uni.lu>
Cc: Stephanie KREIS <stephanie.kreis@uni.lu>; tamara_bintener@hotmail.com; Demetra PHILIPPIDOU <demetra.philippidou@uni.lu>; Christiane WURTH - MARGUE <christiane.margue@uni.lu>; dmgreta@hotmail.it; luca@cancer.dk; Maria Isabel MOSCARDO GARCIA <maria.moscardo@uni.lu>; Rashi HALDER <rashi.halder@uni.lu>; Daniela De Zio (dzio@cancer.dk) <dzio@cancer.dk>; Kulms, Dagmar (Dagmar.Kulms@uniklinikum-dresden.de) <Dagmar.Kulms@uniklinikum-dresden.de>; Maria PACHECO <maria.pacheco@uni.lu>
Subject: Re: Cell Death & Disease paper: co-author Ali Kishk

I confirm.

> Thomas SAUTER <Thomas.Sauter@uni.lu> kirjoitti 29/06/2023 kello 16.27:
>
> Dear co-authors,
>
> Cell Death and Disease requires a written approval from all co-authors of our accepted paper "Metabolic modelling-based in silico drug target prediction identifies six novel repurposable drugs for melanoma"
> That Ali Kishk is added as additional co-author based on his substantial contribution during the revision.
>
> Would you please confirm (or reject)?

A simple “I confirm” with your name would do.

Best regards,
Thomas

Thomas SAUTER

From: Demetra PHILIPPIDOU
Sent: Friday, June 30, 2023 11:08 AM
To: Lasse SINKKONEN; Thomas SAUTER
Cc: Stephanie KREIS; tamara_bintener@hotmail.com; Christiane WURTH - MARGUE; dmgreta@hotmail.it; luca@cancer.dk; Maria Isabel MOSCARDO GARCIA; Rashi HALDER; Daniela De Zio (dzio@cancer.dk); Kulms, Dagmar (Dagmar.Kulms@uniklinikum-dresden.de); Maria PACHECO
Subject: RE: Cell Death & Disease paper: co-author Ali Kishk

I confirm.

Demetra PHILIPPIDOU

Research Support Engineer

UNIVERSITY OF LUXEMBOURG
Faculty of Science, Technology and Medicine
Department of Life Sciences and Medicine
Signal Transduction Laboratory
6, avenue du Swing
L-4367 Belvaux

Tel.: (+352) 46 66 44 **6167**
E-mail: demetra.philippidou@uni.lu

From: Lasse SINKKONEN <lasse.sinkkonen@uni.lu>
Sent: 29 June 2023 17:17
To: Thomas SAUTER <Thomas.Sauter@uni.lu>
Cc: Stephanie KREIS <stephanie.kreis@uni.lu>; tamara_bintener@hotmail.com; Demetra PHILIPPIDOU <demetra.philippidou@uni.lu>; Christiane WURTH - MARGUE <christiane.margue@uni.lu>; dmgreta@hotmail.it; luca@cancer.dk; Maria Isabel MOSCARDO GARCIA <maria.moscardo@uni.lu>; Rashi HALDER <rashi.halder@uni.lu>; Daniela De Zio (dzio@cancer.dk) <dzio@cancer.dk>; Kulms, Dagmar (Dagmar.Kulms@uniklinikum-dresden.de) <Dagmar.Kulms@uniklinikum-dresden.de>; Maria PACHECO <maria.pacheco@uni.lu>
Subject: Re: Cell Death & Disease paper: co-author Ali Kishk

I confirm.

> Thomas SAUTER <Thomas.Sauter@uni.lu> kirjoitti 29/06/2023 kello 16.27:
>
> Dear co-authors,
>
> Cell Death and Disease requires a written approval from all co-authors of our accepted paper "Metabolic modelling-based in silico drug target prediction identifies six novel repurposable drugs for melanoma"
> That Ali Kishk is added as additional co-author based on his substantial contribution during the revision.
>
> Would you please confirm (or reject)?

A simple “I confirm” with your name would do.

Best regards,
Thomas

Thomas SAUTER

From: Greta Del Mistro <dmgreta@hotmail.it>
Sent: Friday, June 30, 2023 10:07 AM
To: Thomas SAUTER
Subject: Re: Cell Death & Disease paper: co-author Ali Kishk

I confirm.
Greta Del Mistro

Il giorno 29 giu 2023, alle ore 16:27, Thomas SAUTER <Thomas.Sauter@uni.lu> ha scritto:

Dear co-authors,

Cell Death and Disease requires a written approval from all co-authors of our accepted paper "Metabolic modelling-based in silico drug target prediction identifies six novel repurposable drugs for melanoma"
That Ali Kishk is added as additional co-author based on his substantial contribution during the revision.

Would you please confirm (or reject)?
A simple "I confirm" with your name would do.

Best regards,
Thomas

Thomas SAUTER

From: Rashi HALDER
Sent: Friday, June 30, 2023 9:58 AM
To: Maria Isabel MOSCARDO GARCIA; Lasse SINKKONEN; Thomas SAUTER
Cc: Stephanie KREIS; tamara_bintener@hotmail.com; Demetra PHILIPPIDOU; Christiane WURTH - MARGUE; dmgreta@hotmail.it; luca@cancer.dk; Daniela De Zio (dzio@cancer.dk); Kulms, Dagmar (Dagmar.Kulms@uniklinikum-dresden.de); Maria PACHECO
Subject: Re: Cell Death & Disease paper: co-author Ali Kishk

I confirm.

Best,
Rashi

From: Maria Isabel MOSCARDO GARCIA <maria.moscardo@uni.lu>
Date: Thursday, June29 2023 at 17:17
To: Lasse SINKKONEN <lasse.sinkkonen@uni.lu>, Thomas SAUTER <Thomas.Sauter@uni.lu>
Cc: Stephanie KREIS <stephanie.kreis@uni.lu>, tamara_bintener@hotmail.com <tamara_bintener@hotmail.com>, Demetra PHILIPPIDOU <demetra.philippidou@uni.lu>, Christiane WURTH - MARGUE <christiane.margue@uni.lu>, dmgreta@hotmail.it <dmgreta@hotmail.it>, luca@cancer.dk <luca@cancer.dk>, Rashi HALDER <rashi.halder@uni.lu>, Daniela De Zio (dzio@cancer.dk) <dzio@cancer.dk>, Kulms, Dagmar (Dagmar.Kulms@uniklinikum-dresden.de) <Dagmar.Kulms@uniklinikum-dresden.de>, Maria PACHECO <maria.pacheco@uni.lu>
Subject: Re: Cell Death & Disease paper: co-author Ali Kishk

I confirm.

Best regards,

Maria

--
Maria Isabel Moscardó García
PhD Candidate
Systems Control Group
Luxembourg Centre for Systems Biomedicine (LCSB)
University of Luxembourg
6, Avenue du Swing
L-4367 Belvaux

From: Lasse SINKKONEN <lasse.sinkkonen@uni.lu>
Date: Thursday, 29 June 2023 at 17:16
To: Thomas SAUTER <Thomas.Sauter@uni.lu>
Cc: Stephanie KREIS <stephanie.kreis@uni.lu>, tamara_bintener@hotmail.com <tamara_bintener@hotmail.com>, Demetra PHILIPPIDOU <demetra.philippidou@uni.lu>, Christiane WURTH - MARGUE <christiane.margue@uni.lu>, dmgreta@hotmail.it <dmgreta@hotmail.it>, luca@cancer.dk <luca@cancer.dk>, Maria Isabel MOSCARDO GARCIA <maria.moscardo@uni.lu>, Rashi HALDER <rashi.halder@uni.lu>, Daniela De Zio (dzio@cancer.dk) <dzio@cancer.dk>, Kulms, Dagmar (Dagmar.Kulms@uniklinikum-dresden.de) <Dagmar.Kulms@uniklinikum-dresden.de>, Maria PACHECO

<maria.pacheco@uni.lu>
Subject: Re: Cell Death & Disease paper: co-author Ali Kishk

I confirm.

Thomas SAUTER <Thomas.Sauter@uni.lu> kirjoitti 29/06/2023 kello 16.27:

Dear co-authors,

Cell Death and Disease requires a written approval from all co-authors of our accepted paper "Metabolic modelling-based in silico drug target prediction identifies six novel repurposable drugs for melanoma"
That Ali Kishk is added as additional co-author based on his substantial contribution during the revision.

Would you please confirm (or reject)?
A simple "I confirm" with your name would do.

Best regards,
Thomas

Thomas SAUTER

From: Tamara Bintener <tamara.bintener@gmail.com>
Sent: Thursday, June 29, 2023 8:24 PM
To: Thomas SAUTER
Subject: Re: Cell Death & Disease paper: co-author Ali Kishk

Hello,

That's great news!

I confirm.

Best,

Tamara Bintener

On Thu, 29 Jun 2023, 16:27 Thomas SAUTER, <Thomas.Sauter@uni.lu> wrote:

> Dear co-authors,
>
> Cell Death and Disease requires a written approval from all co-authors of our accepted paper
>
> "Metabolic modelling-based in silico drug target prediction identifies six novel repurposable drugs for melanoma"
>
> That Ali Kishk is added as additional co-author based on his substantial contribution during the revision.
>
> Would you please confirm (or reject)?
>
> A simple “I confirm” with your name would do.
>
> Best regards,
>
> Thomas

Thomas SAUTER

From: Stephanie KREIS
Sent: Thursday, June 29, 2023 5:39 PM
To: Lasse SINKKONEN; Thomas SAUTER
Subject: RE: Cell Death & Disease paper: co-author Ali Kishk

I confirm.
Best,
stephanie

Prof. Dr. Stephanie Kreis
Associate Professor
Co-Head Signal Transduction Group, Department of Life Science and Medicine (DLSM)
Head of Doctoral School in Science and Engineering (DSSE)
UNIVERSITY OF LUXEMBOURG

BELVAL CAMPUS
Biotech 2 / 4th floor / office 424
6, Avenue du Swing / L-4367 Belvaux
Phone: +352 466644 6884
Stephanie.Kreis@uni.lu / www.uni.lu

From: Lasse SINKKONEN <lasse.sinkkonen@uni.lu>
Sent: Thursday, June 29, 2023 5:17 PM
To: Thomas SAUTER <Thomas.Sauter@uni.lu>
Cc: Stephanie KREIS <stephanie.kreis@uni.lu>; tamara_bintener@hotmail.com; Demetra PHILIPPIDOU <demetra.philippidou@uni.lu>; Christiane WURTH - MARGUE <christiane.margue@uni.lu>; dmgreta@hotmail.it; luca@cancer.dk; Maria Isabel MOSCARDO GARCIA <maria.moscardo@uni.lu>; Rashi HALDER <rashi.halder@uni.lu>; Daniela De Zio (dzio@cancer.dk) <dzio@cancer.dk>; Kulms, Dagmar (Dagmar.Kulms@uniklinikum-dresden.de) <Dagmar.Kulms@uniklinikum-dresden.de>; Maria PACHECO <maria.pacheco@uni.lu>
Subject: Re: Cell Death & Disease paper: co-author Ali Kishk

I confirm.

> Thomas SAUTER <Thomas.Sauter@uni.lu> kirjoitti 29/06/2023 kello 16.27:
>
> Dear co-authors,
>
> Cell Death and Disease requires a written approval from all co-authors of our accepted paper "Metabolic modelling-based in silico drug target prediction identifies six novel repurposable drugs for melanoma"
> That Ali Kishk is added as additional co-author based on his substantial contribution during the revision.
>
> Would you please confirm (or reject)?
> A simple “I confirm” with your name would do.
>
> Best regards,
> Thomas

Thomas SAUTER

From: Christiane WURTH - MARGUE
Sent: Thursday, June 29, 2023 5:36 PM
To: Thomas SAUTER
Subject: Re: Cell Death & Disease paper: co-author Ali Kishk

I confirm
Christiane Margue

From: Thomas SAUTER <Thomas.Sauter@uni.lu>
Sent: Thursday, June 29, 2023 16:26
To: Stephanie KREIS <stephanie.kreis@uni.lu>; tamara_bintener@hotmail.com <tamara_bintener@hotmail.com>; Demetra PHILIPPIDOU <demetra.philippidou@uni.lu>; Christiane WURTH - MARGUE <christiane.margue@uni.lu>; dmgreta@hotmail.it <dmgreta@hotmail.it>; luca@cancer.dk <luca@cancer.dk>; Maria Isabel MOSCARDO GARCIA <maria.moscardo@uni.lu>; Rashi HALDER <rashi.halder@uni.lu>; Lasse SINKKONEN <lasse.sinkkonen@uni.lu>; Daniela De Zio (dzio@cancer.dk) <dzio@cancer.dk>; Kulms, Dagmar (Dagmar.Kulms@uniklinikum-dresden.de) <Dagmar.Kulms@uniklinikum-dresden.de>; Maria PACHECO <maria.pacheco@uni.lu>
Subject: Cell Death & Disease paper: co-author Ali Kishk

Dear co-authors,

Cell Death and Disease requires a written approval from all co-authors of our accepted paper
"Metabolic modelling-based in silico drug target prediction identifies six novel repurposable drugs for melanoma"
That Ali Kishk is added as additional co-author based on his substantial contribution during the revision.

Would you please confirm (or reject)?
A simple “I confirm” with your name would do.

Best regards,
Thomas

Thomas SAUTER

From: Maria Isabel MOSCARDO GARCIA
Sent: Thursday, June 29, 2023 5:18 PM
To: Lasse SINKKONEN; Thomas SAUTER
Cc: Stephanie KREIS; tamara_bintener@hotmail.com; Demetra PHILIPPIDOU; Christiane WURTH - MARGUE; dmgreta@hotmail.it; luca@cancer.dk; Rashi HALDER; Daniela De Zio (dzio@cancer.dk); Kulms, Dagmar (Dagmar.Kulms@uniklinikum-dresden.de); Maria PACHECO
Subject: Re: Cell Death & Disease paper: co-author Ali Kishk

I confirm.

Best regards,

Maria

--
Maria Isabel Moscardó García
PhD Candidate
Systems Control Group
Luxembourg Centre for Systems Biomedicine (LCSB)
University of Luxembourg
6, Avenue du Swing
L-4367 Belvaux

From: Lasse SINKKONEN <lasse.sinkkonen@uni.lu>
Date: Thursday, 29 June 2023 at 17:16
To: Thomas SAUTER <Thomas.Sauter@uni.lu>
Cc: Stephanie KREIS <stephanie.kreis@uni.lu>, tamara_bintener@hotmail.com <tamara_bintener@hotmail.com>, Demetra PHILIPPIDOU <demetra.philippidou@uni.lu>, Christiane WURTH - MARGUE <christiane.margue@uni.lu>, dmgreta@hotmail.it <dmgreta@hotmail.it>, luca@cancer.dk <luca@cancer.dk>, Maria Isabel MOSCARDO GARCIA <maria.moscardo@uni.lu>, Rashi HALDER <rashi.halder@uni.lu>, Daniela De Zio (dzio@cancer.dk) <dzio@cancer.dk>, Kulms, Dagmar (Dagmar.Kulms@uniklinikum-dresden.de) <Dagmar.Kulms@uniklinikum-dresden.de>, Maria PACHECO <maria.pacheco@uni.lu>
Subject: Re: Cell Death & Disease paper: co-author Ali Kishk

I confirm.

> Thomas SAUTER <Thomas.Sauter@uni.lu> kirjoitti 29/06/2023 kello 16.27:
>
> Dear co-authors,
>
> Cell Death and Disease requires a written approval from all co-authors of our accepted paper
> "Metabolic modelling-based in silico drug target prediction identifies six novel repurposable drugs for melanoma"
> That Ali Kishk is added as additional co-author based on his substantial contribution during the revision.

Would you please confirm (or reject)?
A simple “I confirm” with your name would do.

Best regards,
Thomas

Thomas SAUTER

**From:**	Lasse SINKKONEN
**Sent:**	Thursday, June 29, 2023 5:17 PM
**To:**	Thomas SAUTER
**Cc:**	Stephanie KREIS; tamara_bintener@hotmail.com; Demetra PHILIPPIDOU; Christiane WURTH - MARGUE; dmgreta@hotmail.it; luca@cancer.dk; Maria Isabel MOSCARDO GARCIA; Rashi HALDER; Daniela De Zio (dzio@cancer.dk); Kulms, Dagmar (Dagmar.Kulms@uniklinikum-dresden.de); Maria PACHECO
**Subject:**	Re: Cell Death & Disease paper: co-author Ali Kishk

I confirm.

Thomas SAUTER <Thomas.Sauter@uni.lu> kirjoitti 29/06/2023 kello 16.27:

Dear co-authors,

Cell Death and Disease requires a written approval from all co-authors of our accepted paper "Metabolic modelling-based in silico drug target prediction identifies six novel repurposable drugs for melanoma"
That Ali Kishk is added as additional co-author based on his substantial contribution during the revision.

Would you please confirm (or reject)?
A simple “I confirm” with your name would do.

Best regards,
Thomas

Thomas SAUTER

From: dzio@cancer.dk on behalf of Daniela De Zio <dzio@cancer.dk>
Sent: Thursday, June 29, 2023 4:43 PM
To: Thomas SAUTER; Stephanie KREIS; tamara_bintener@hotmail.com; Demetra PHILIPPIDOU; Christiane WURTH - MARGUE; dmgreta@hotmail.it; Luca Di Leo; Maria Isabel MOSCARDO GARCIA; Rashi HALDER; Lasse SINKKONEN; Kulms, Dagmar (Dagmar.Kulms@uniklinikum-dresden.de); Maria PACHECO
Subject: Re: Cell Death & Disease paper: co-author Ali Kishk

I confirm

Daniela De Zio
Associate Professor
Melanoma Research Team

Danish Cancer Institute
Strandboulevarden 49
DK-2100 Copenhagen
Telephone: +45 35 25 74 03

Danish Cancer Society

www.cancer.dk | Vores privatlivspolitik

From: Thomas SAUTER <Thomas.Sauter@uni.lu>
Date: Thursday, 29 June 2023 at 16.27
To: Stephanie KREIS <stephanie.kreis@uni.lu>, tamara_bintener@hotmail.com <tamara_bintener@hotmail.com>, Demetra PHILIPPIDOU <demetra.philippidou@uni.lu>, Christiane WURTH - MARGUE <christiane.margue@uni.lu>, dmgreta@hotmail.it <dmgreta@hotmail.it>, Luca Di Leo <luca@cancer.dk>, Maria Isabel MOSCARDO GARCIA <maria.moscardo@uni.lu>, Rashi HALDER <rashi.halder@uni.lu>, Lasse SINKKONEN <lasse.sinkkonen@uni.lu>, Daniela De Zio <dzio@cancer.dk>, Kulms, Dagmar (Dagmar.Kulms@uniklinikum-dresden.de) <Dagmar.Kulms@uniklinikum-dresden.de>, Maria PACHECO <maria.pacheco@uni.lu>
Subject: Cell Death & Disease paper: co-author Ali Kishk

Dear co-authors,

Cell Death and Disease requires a written approval from all co-authors of our accepted paper
"Metabolic modelling-based in silico drug target prediction identifies six novel repurposable drugs for melanoma"
That Ali Kishk is added as additional co-author based on his substantial contribution during the revision.

Would you please confirm (or reject)?
A simple “I confirm” with your name would do.

Best regards,
Thomas

Thomas SAUTER

From: Luca Di Leo <luca@cancer.dk>
Sent: Thursday, June 29, 2023 4:55 PM
To: Thomas SAUTER
Subject: Re: Cell Death & Disease paper: co-author Ali Kishk

I confirm

Luca Di Leo, PhD

Danish Cancer Society Research Center
Melanoma Research Team
Cell Stress and Survival

Strandboulevarden 49
2100 København Ø
Denmark

Phone: +45 35 25 74 04
Mobile: +45 31 32 05 91
e-mail: luca@cancer.dk

From: Thomas SAUTER <Thomas.Sauter@uni.lu>
Sent: Thursday, June 29, 2023 4:26:59 PM
To: Stephanie KREIS <stephanie.kreis@uni.lu>; tamara_bintener@hotmail.com <tamara_bintener@hotmail.com>; Demetra PHILIPPIDOU <demetra.philippidou@uni.lu>; Christiane WURTH - MARGUE <christiane.margue@uni.lu>; dmgreta@hotmail.it <dmgreta@hotmail.it>; Luca Di Leo <luca@cancer.dk>; Maria Isabel MOSCARDO GARCIA <maria.moscardo@uni.lu>; Rashi HALDER <rashi.halder@uni.lu>; Lasse SINKKONEN <lasse.sinkkonen@uni.lu>; Daniela De Zio <dzio@cancer.dk>; Kulms, Dagmar (Dagmar.Kulms@uniklinikum-dresden.de) <Dagmar.Kulms@uniklinikum-dresden.de>; Maria PACHECO <maria.pacheco@uni.lu>
Subject: Cell Death & Disease paper: co-author Ali Kishk

Dear co-authors,

Cell Death and Disease requires a written approval from all co-authors of our accepted paper
"Metabolic modelling-based in silico drug target prediction identifies six novel repurposable drugs for melanoma"
That Ali Kishk is added as additional co-author based on his substantial contribution during the revision.

Would you please confirm (or reject)?
A simple “I confirm” with your name would do.

Best regards,
Thomas

Thomas SAUTER

From: Maria PACHECO
Sent: Thursday, June 29, 2023 4:35 PM
To: Thomas SAUTER; Stephanie KREIS; tamara_bintener@hotmail.com; Demetra PHILIPPIDOU; Christiane WURTH - MARGUE; dmgreta@hotmail.it; luca@cancer.dk; Maria Isabel MOSCARDO GARCIA; Rashi HALDER; Lasse SINKKONEN; Daniela De Zio (dzio@cancer.dk); Kulms, Dagmar (Dagmar.Kulms@uniklinikum-dresden.de)
Subject: Re: Cell Death & Disease paper: co-author Ali Kishk

I confirm.

Maria Pires Pacheco
Post doctoral researcher
Department of Life Sciences and Medicine

UNIVERSITÉ DU LUXEMBOURG

CAMPUS BELVAL
2, avenue de l'Université
L-4365 Esch-sur-Alzette

From: Thomas SAUTER <Thomas.Sauter@uni.lu>
Sent: Thursday, June 29, 2023 16:26
To: Stephanie KREIS <stephanie.kreis@uni.lu>; tamara_bintener@hotmail.com <tamara_bintener@hotmail.com>; Demetra PHILIPPIDOU <demetra.philippidou@uni.lu>; Christiane WURTH - MARGUE <christiane.margue@uni.lu>; dmgreta@hotmail.it <dmgreta@hotmail.it>; luca@cancer.dk <luca@cancer.dk>; Maria Isabel MOSCARDO GARCIA <maria.moscardo@uni.lu>; Rashi HALDER <rashi.halder@uni.lu>; Lasse SINKKONEN <lasse.sinkkonen@uni.lu>; Daniela De Zio (dzio@cancer.dk) <dzio@cancer.dk>; Kulms, Dagmar (Dagmar.Kulms@uniklinikum-dresden.de) <Dagmar.Kulms@uniklinikum-dresden.de>; Maria PACHECO <maria.pacheco@uni.lu>
Subject: Cell Death & Disease paper: co-author Ali Kishk

Dear co-authors,

Cell Death and Disease requires a written approval from all co-authors of our accepted paper
"Metabolic modelling-based in silico drug target prediction identifies six novel repurposable drugs for melanoma"
That Ali Kishk is added as additional co-author based on his substantial contribution during the revision.

Would you please confirm (or reject)?
A simple "I confirm" with your name would do.

Best regards,
Thomas

From: Kulms, Dagmar <Dagmar.Kulms@ukdd.de>
Sent: Thursday, June 29, 2023 4:32 PM
To: Thomas SAUTER
Subject: AW: Cell Death & Disease paper: co-author Ali Kishk

Dear Thomas,

I confirm!

Best regards,
Dagmar Kulms

____
Prof. Dr.rer.nat. Dagmar Kulms
Experimental Dermatology
National Center of Tumor Diseases, NCT
Medical Faculty, TU-Dresden
Fetscherstraße 74, House 136
01307 Dresden
Phone: +49 351 45818973
email: dagmar.kulms@ukdd.de
www.uniklinikum-dresden.de

Von: Thomas SAUTER <Thomas.Sauter@uni.lu>
Gesendet: Donnerstag, 29. Juni 2023 16:27
An: Stephanie KREIS <stephanie.kreis@uni.lu>; tamara_bintener@hotmail.com; Demetra PHILIPPIDOU <demetra.philippidou@uni.lu>; Christiane WURTH - MARGUE <christiane.margue@uni.lu>; dmgreta@hotmail.it; luca@cancer.dk; Maria Isabel MOSCARDO GARCIA <maria.moscardo@uni.lu>; Rashi HALDER <rashi.halder@uni.lu>; Lasse SINKKONEN <lasse.sinkkonen@uni.lu>; Daniela De Zio (dzio@cancer.dk) <dzio@cancer.dk>; Kulms, Dagmar <Dagmar.Kulms@ukdd.de>; Maria PACHECO <maria.pacheco@uni.lu>
Betreff: Cell Death & Disease paper: co-author Ali Kishk

Dear co-authors,

Cell Death and Disease requires a written approval from all co-authors of our accepted paper "Metabolic modelling-based in silico drug target prediction identifies six novel repurposable drugs for melanoma" That Ali Kishk is added as additional co-author based on his substantial contribution during the revision.

Would you please confirm (or reject)?
A simple “I confirm” with your name would do.

Best regards,
Thomas

Thomas SAUTER

From: Thomas SAUTER
Sent: Thursday, June 29, 2023 4:27 PM
To: Stephanie KREIS; tamara_bintener@hotmail.com; Demetra PHILIPPIDOU; Christiane WURTH - MARGUE; dmgreta@hotmail.it; luca@cancer.dk; Maria Isabel MOSCARDO GARCIA; Rashi HALDER; Lasse SINKKONEN; Daniela De Zio (dzio@cancer.dk); Kulms, Dagmar (Dagmar.Kulms@uniklinikum-dresden.de); Maria PACHECO
Subject: Cell Death & Disease paper: co-author Ali Kishk

Dear co-authors,

Cell Death and Disease requires a written approval from all co-authors of our accepted paper
"Metabolic modelling-based in silico drug target prediction identifies six novel repurposable drugs for melanoma"
That Ali Kishk is added as additional co-author based on his substantial contribution during the revision.

Would you please confirm (or reject)?
A simple “I confirm” with your name would do.

Best regards,
Thomas
